# Quercetin prophylaxis protects the kidneys by modulating the renin–angiotensin–aldosterone axis under acute hypobaric hypoxic stress

**DOI:** 10.1038/s41598-024-58134-3

**Published:** 2024-03-31

**Authors:** Vaishnavi Rathi, Sarada S. K. Sagi, Amit Kumar Yadav, Manoj Kumar, Rajeev Varshney

**Affiliations:** 1 Defence Institute of Physiology and Allied Sciences, DRDO, Lucknow Road, Timarpur, New Delhi, 110054 India; 2 Department of Biophysics, All India Institute of Medical Science, Delhi, India

**Keywords:** Molecular biology, Proteomics

## Abstract

The study presented here aims at assessing the effects of hypobaric hypoxia on RAAS pathway and its components along with mitigation of anomalies with quercetin prophylaxis. One hour prior to hypobaric hypoxia exposure, male SD rats were orally supplemented with quercetin (50 mg/kg BW) and acetazolamide (50 mg/kg BW) and exposed them to 25,000 ft. (7,620 m) in a simulated environmental chamber for 12 h at 25 ± 2 °C. Different biochemical parameters like renin activity, aldosterone, angiotensin I, ACE 2 were determined in plasma. As a conventional response to low oxygen conditions, oxidative stress parameters (ROS and MDA) were elevated along with suppressed antioxidant system (GPx and catalase) in plasma of rats. Quercetin prophylaxis significantly down regulated the hypoxia induced oxidative stress by reducing plasma ROS & MDA levels with efficient enhancement of antioxidants (GPx and Catalase). Further, hypoxia mediated regulation of renin and ACE 2 proves the outstanding efficacy of quercetin in repudiating altercations in RAAS cascade due to hypobaric hypoxia. Furthermore, differential protein expression of HIF-1α, NFκB, IL-18 and endothelin-1 analyzed by western blotting approves the biochemical outcomes and showed that quercetin significantly aids in the reduction of inflammation under hypoxia. Studies conducted with Surface Plasmon Resonance demonstrated a binding among quercetin and ACE 2 that indicates that this flavonoid might regulate RAAS pathway via ACE 2. Henceforth, the study promotes the prophylaxis of quercetin for the better adaptability under hypobaric hypoxic conditions via modulating the RAAS pathway.

## Introduction

Exposure to hypobaric hypoxic environment such as high altitude regions induces a rise in blood pressure, which possess a direct connection with renin–angiotensin–aldosterone system (RAAS). RAAS is one of the major regulatory pathways involved in acclimatization and maintenance of blood pressure in sojourners ascending to mountains. Angiotensin II (Ang II), a mature octa peptide serves as the final effector presser component of the pathway formed from the exopeptidase activity of Angiotensin Converting Enzyme (ACE) on carboxy-terminal of Angiotensin I (Ang I). Localized on the plasma membrane of various cell types, ACE is involved in the production of Ang II in lungs, kidneys, brain etc^1^. Ang II stimulates adrenal cortex to secrete aldosterone that increases the sodium reabsorption by stimulating proximal tubules of kidneys^2,3^. Angiotensin Converting Enzyme 2 (ACE 2), a sequentially similar novel homologue of ACE, is known for its contradictory action on RAAS axis^1^.

Interplay of escalated blood flow with declined metabolism compensates for the lack of oxygen at high altitude and prevent tissue damage. Kidneys along with carotid bodies functions as the main hypoxia sensors which elicit reflex responses that include elevated sympathetic activity, vascular resistance and arterial blood pressure^4,5^. Stimulation of carotid bodies under hypoxia results in activation of peripheral chemoreceptors that leads to elevated heart rate with an upsurge in blood pressure and cardiac output in response to direct vasodilator effect of hypoxia^6–8^. However, this increase in heart rate is due to withdrawal in parasympathetic activity rather than activation of sympathetic system which has been evidently proven in earlier researches. Several studies witnessed an uninterrupted higher heart rate in spite of treatment with beta-adrenergic blockers^9,10^. Therefore, kidneys, the active members of the ‘*hypoxia sensing panel*’ plays an intricate role in regulation of blood pressure, acclimatization and development of high altitude maladies. Renin being an important renal enzyme and foremost member of RAAS acts as the chief regulator of blood pressure and thus, we hypothesize that RAAS might be the appropriate system to unravel the underlying mechanism of multiple complications encountered at high altitude. Although, until now, its role in this setting remain questionable but hypoxia might have a direct effect on RAAS which may play a crucial role in pathogenesis of acute mountain sickness (AMS) and high altitude renal syndrome (HARS). Furthermore, the mechanism of natriuretic and diuretic effect under hypoxic conditions is not well-known till date. Several studies have suggested that peripheral chemo reflex holds the accountability for high altitude water and sodium diuresis^11–13^. Previously available data demonstrates a decline in sodium retaining hormones, particularly pertaining to renin–angiotensin–aldosterone system^14–16^.

ACE 2, a 42% homolog of ACE and a monopeptidase with enzymatic preference for hydrophobic residues of Ang II at C-terminus, aids in the conversion of Ang II to Angiotensin -(1–7) (Ang-(1–7)). Animal and human researches demonstrates the pro-inflammatory response of Ang II in arteries, kidney and heart by regulation of cytokine and chemokine expression^14^. Antagonistic behavior of Ang-(1–7) with respect to Ang II has been proven in numerous experimental studies. This implies that Ang-(1–7) acts as the anti-inflammatory component of this system which has cardio-renal protecting abilities^17–19^. Thus, emphasis can be drawn upon the pro-inflammatory and pro-fibrotic effects of RAAS at cellular and molecular level whereas ACE2 deviate this pathway to anti-inflammatory axis by forming Ang-(1–7).

Hence, this study was programmed to speculate the effects of simulated hypobaric hypoxic conditions on the components of RAAS and their modulation by pharmacological intervention with quercetin. Through this study, we intend to evaluate the efficacy of quercetin as a nephroprotective molecule against high altitude induced renal impairment. We have studied this mechanism in kidneys of the rats, due to the compensatory mechanism to be taken care by the kidney upon immediate exposure to hypobaric hypoxia for acclimatization. The RAAS pathway begins with renin released by kidneys in to the systemic circulation and modulates the RAAS components at several stages ultimately causing increased vasoconstriction leading to increased blood pressure there by acclimatization is not attained properly in order to survive best at high altitude. So we have targeted the renin secreted by kidneys and the ACE2 enzyme that changes the pro-inflammatory to –anti-inflammatory axis as main concern under this study. Previous studies showed that angiotensin inhibitors including bioflavonoids not only enhances the renal functions under diseased conditions but also have renal protective attributes^20^. Among innumerable plant-based foods available today, flavonoids are the chief dietary constituents ubiquitously present in fruits, vegetables and beverages^21,22^. Quercetin, a well-known phyto-flavonoid, with antioxidant and anti-inflammatory properties quenches free radicals and serves as a protective molecule against tissue damage. Thus, its binding with numerous enzymatic and non-enzymatic targets in renin–angiotensin–aldosterone axis is one of the most important characteristic feature^23^. Hence, the present article attempts to find out the binding efficacy of quercetin with ACE 2 using Surface Plasmon Resonance (SPR).

Apart from RAAS, maintenance of electrolytes is another crucial function of kidneys which are directly associated with high altitude adaptive physiology. Renal excretory rate of sodium and potassium under high altitude hypoxia is controversial. Ascent to high altitudes results in decreased sodium concentrations in plasma whereas potassium levels remains unchanged as stated by Singh et al^24^. However, contradicting studies are available as well. Hence, this study attempts to figure out the possible pattern of sodium, potassium and chloride ions under hypobaric hypoxia along with regulation of these electrolytes by intervention with quercetin and acetazolamide.

The study presented here investigates the prophylactic efficacy of quercetin in comparison to acetazolamide, which is a commonly administered drug to high altitude sojourners. Acetazolamide is the first-line of prophylaxis, FDA approved drug for AMS that works at the level of kidneys and causes bicarbonate diuresis and metabolic acidosis, promoting ventilatory acclimatization to hypoxia^25^.

## Material and methods

All methods are reported in accordance with ARRIVE guidelines (https://arriveguidelines.org).

### Chemicals and reagents

Quercetin and 2,7-Dichlorofluroscein diacetate (DCFH-DA) were procured from Sigma Aldrich (St. Louis MO, USA), Dimethylsulphoxide (DMSO) from Sisco Research Laboratory (SRL, Maharashtra) along with Thiobarbituric acid (TBA) and Tricarboxylic acid (TCA). 5′5′-dithio-bis-(2-nitro-benzoic acid) (DTNB) from Sigma Aldrich. Acetazolamide was procured from Sun Pharma Laboratories limited. All the other chemicals and reagents were of analytical grade.

### Drug preparation

Drugs quercetin (50 mg/kg BW) and acetazolamide (50 mg/kg BW) were freshly prepared by dissolving into a vehicle i.e., 0.5% DMSO and supplemented orally to Sprague Dawley (SD) rats 1 h prior to the hypobaric hypoxia exposure. Quercetin 50 mg/Kg BW dose was opted, based on the dose standardization studies carried out in our laboratory^26^. Dosage of acetazolamide was given on the basis of the previous literature^27^.

### Experimental animals

Male Sprague Dawley rats of weight 180–200 g were obtained from central animal facility of DIPAS-DRDO, Delhi, India. Animals were kept in experimentally designed polypropylene cages of dimension 32in. × 24in. × 16in. provided with standard conditions (12 light/dark cycle, 25 ± 2 °C temperature & 55 ± 5% relative humidity) and availability of animal chow and water ad libitum. All the animal studies and protocols are in accordance with standards provided in the guide for the Care and Use of Laboratory Animals (National Academy of Sciences, Washington, DC). Protocols involving animal studies were reviewed and sanctioned by the Institutional Animal Ethics Committee (IAEC), DIPAS, Delhi, India, accredited to Committee for the Purpose of Control and Supervision of Experiments on Animals (CPCSEA), Government of India (IAEC No.: DIPAS/IAEC/2017/19/EXT/22).

### Experimental protocol

The experiments were performed with six groups (*n* = 6) of Sprague Dawley rats. (i) Group:1 include normoxia (N) considered as control that received only vehicle (ii) Group 2 : Hypoxia (H) received only vehicle and was exposed to hypoxia for 12 h (iii) Group 3: Normoxia + Quercetin (NQ) was supplemented with quercetin (50 mg/Kg BW) without hypoxia exposure (iv) Group 4: Hypoxia + Quercetin (HQ) received quercetin (50 mg/Kg BW) 1 h prior to hypoxia exposure for 12 h (v) Group 5: Normoxia + Acetazolamide (NA) was supplemented with acetazolamide (50 mg/Kg BW) without hypoxia exposure (vi) Group 6 : Hypoxia + Acetazolamide (HA) received acetazolamide (50 mg/Kg BW) 1 h prior to hypoxia exposure for 12 h. Efficacy of different doses of quercetin (25, 50, 100 and 200 mg/Kg BW) was assessed in previous experiments from our lab and 50 mg/Kg BW was found to be the most efficient dose which showed decreased oxidative stress i.e. Reactive Oxygen Species (ROS) and Malondialdehyde (MDA) along with tissue injury marker i.e. Lactate Dehydrogenase (LDH) significantly. This dose also enhanced the antioxidant activity i.e. Glutathione reductase (GSH) followed by improved renal function parameters i.e. creatinine, Blood Urea Nitrogen (BUN) and uric acid under hypoxic stress among other tested doses^26^.

### Hypoxia exposure

The rats were exposed to hypobaric hypoxia in a simulated hypoxia chamber (Matrix, India) for 12 h at an altitude of 25,000 ft. (7620 m). Standardization of time duration was performed in our previous experiments which showed that, 12 h duration (among the tested durations 1 h, 3 h, 6 h, 12 h, 24 h and 48 h) of hypoxia exposure was the optimum time, at which maximum renal damage had occurred^26^. Internal hypobaric hypoxia chamber pressure was maintained at (280 mm Hg) with fresh air flushing at the rate of 4 lit/h along with the relative humidity of 55 ± 5%. Further, the partial pressure of oxygen (pO_2_) in control rats was observed to be around 96 ± 2 mmHg, whereas in hypoxia exposed rats pO_2_ was around 35 ± 2 mmHg. This indicates that the animals were exposed to low barometric pressure at high altitude. Standard animal chow and water was made available ad libitum during hypoxia exposure to rats. All animal experiments were performed with utmost care to minimize the sufferings on rats.

### Method of sacrifice

After 12 h of hypobaric hypoxia exposure, rats were injected intraperitoneally (IP) with anesthetic agents (Ketamine-Xylazine cocktail with 100:20 ratio). Animals were sacrificed after appropriate sedation.

### Preparation of sample:

Animals were perfused with chilled 1X PBS at the time of dissection. Kidney tissues were washed with 1X PBS and stored at − 80 °C for protein expression studies. Blood was collected from left ventricle of the heart; the plasma was separated and kept at − 80 °C for further biochemical analysis. Urine samples were collected by bladder puncture during dissection of the animals under proper sedation. Urine samples were centrifuged immediately (300 × g for 5 min) for removal of debris and stored at − 80 °C.

### Biochemical assays

#### Measurement of oxidative stress

Estimation of reactive oxygen species (ROS) generation in plasma was carried out using 2, 7-dichlorofluorescein diacetate (DCF-DA) assay in normoxic and hypoxia exposed rats^28,29^. DCFH-DA along with potassium dihydrogen buffer was added to plasma prior to incubation of 15 min at room temperature in dark. Fluorescence emitted by DCF formed as a result of oxidation of DCFH-DA in the presence of ROS was measured by spectrophotometrically (Synergy H1, Biotek, Germany) at an excitation of 485 nm and emission of 530 nm.

#### Malondialdedhyde (MDA) estimation

The assay involves condensation reaction of two molecules of TBA with one molecule of MDA which is formed as a byproduct of lipid peroxidation in plasma of tested groups. The method consists of incubating samples (plasma) at 80 °C with TBA, TCA and HCl for 1 h, the reaction mixture was further centrifuged at 3000 rpm for 10 min at 4 °C. The absorbance of the MDA-TBA adduct formed was measured spectrophotometrically at 532 nm^30,31^.

#### GPx and catalase activity

The antioxidant activity of GPx and catalase were carried out in plasma by assay kits provided by manufacturer cat no.: EGPx-100, CSB-E13439r (Cusabio, USA).

#### Plasma renin activity

Estimation of plasma renin activity was carried out by commercially available Plasma Renin Activity (PRA) ELISA (LDN- Immunoassays and Services), as per the procedure elaborated in manufacturer’s guidelines.

#### Angiotensin I and II

Evaluation of Angiotensin I and II in plasma of normoxia control, hypoxia and drug treated groups were performed on the basis of instructions provided by the manufacturer for rat angiotensin I (Sunlong Biotech, China) and II (Cusabio, USA) ELISA kits.

#### Angiotensin converting enzyme 2

Quantitative estimation of ACE 2 enzyme was done in plasma samples of normoxia control, hypoxic and quercetin and acetazolamide pretreated rats. The assay was performed as per manufacturer’s guidelines provided with the ELISA kit (Cusabio, USA).

#### Aldosterone

Aldosterone is a steroidal hormone released by zona glomerulosa cells of adrenal cortex of kidney. It functions in reabsorption and secretion of Na^+^ and K^+^ ions, respectively thus maintains the electrolyte and fluid balance. Quantitative estimation of aldosterone was performed in plasma and urine in accordance with the manufacturer’s guidelines provided along with assay kit (Diagnostics Biochem Inc., Canada).

### Protein expression studies

Nuclear and cytoplasmic extracts were prepared using lysis (10 mM HEPES, 1.5 mM MgCl_2_, 10 mM KCl) and extraction buffers (20 mM HEPES, 1.5 mM MgCl_2_, 0.42 M NaCl, 0.2 mM EDTA, 25% glycerol). Protein quantification was estimated by Lowry’s method as mentioned elsewhere^26^. Western blot analysis was carried out for separation and identification of Hypoxia-Inducible Factor-1 alpha (HIF-1α), Nuclear Factor kappa B (NFκB), histone-3, from nuclear fractions of kidney tissues. Whereas, interleukin-18 (IL-18), endothelin-1 (ET-1) and α-tubulin protein expressions were determined from cytoplasmic extracts of kidneys of rats exposed to hypoxia.

Histone-3 and α-tubulin protein expressions were used as housekeeping genes to represent nuclear and cytoplasmic protein expressions respectively. The protein in kidney samples were separated using 10% and 12% SDS-PAGE respectively. The separated proteins were then, electro-blotted to the nitrocellulose membranes and blocked with 5% BSA dissolved in 1X PBST (pH 7.4) at RT for 1 h with gentle shaking. Membranes were then washed and incubated with respective primary antibodies (Santa Cruz Biotechnology (HIF-1α, NFκB, IL-18, endothelin-1; 1:1000 dilution) over night at 4 °C. After 4–5 washings with PBST (Tween 0.1%), the membranes were probed with HRP-conjugated secondary antibodies (Santa Cruz Biotechnology 1: 20,000 dilution) at RT for 2 h. The blots were then thoroughly washed (4–5 times) with PBST. The membranes were developed using chemiluminescent peroxidase substrate (Luminata forte, Millipore U.S.A) and the bands were visualized in Chemidoc (UVP, Cambridge, U.K). The optical density (OD) of the bands were quantified using lab works software (UVP-Bio Imaging systems, CA).

#### Surface plasmon resonance spectrometry

Binding affinity of analytes (quercetin and acetazolamide) towards the ligand (ACE 2) was analyzed using Surface Plasmon Resonance (SPR) spectroscopy (Autolab Esprit) which functions on the principle of Taylor Dispersion Analysis (TDA). According to TDA, concentration of analyte changes uniformly and gradually, however the association of analytes with the respective ligand is represented in a sigmoidal gradient pattern. The variable concentrations of analyte dissolved in 0.5% Dimethyl Sulphoxide (DMSO) and running buffer (HEPES = 10 mM, NaCl = 150 mM, Tween 20 = 0.005%, EDTA = 1.26 mM) ranging from 1.5 to 100 μM was run against ligand (ACE 2) prepared in sodium acetate (pH∼5.0, 10 mM) which was coated on a gold chip^32^.

#### Ion analysis

Ions like Sodium (Na^+^), Potassium (K^+^) and Chloride (Cl^−^) along with Hemoglobin (Hb) were measured via single use cartridge (CHEM 8 +) based *i-STAT* 1 blood analyzer (Abbott, U.S.) Na^+^, K^+^ and Cl^−^ ions were expressed in mmol/L whereas Hb was expressed in mg/dl.

#### Statistical analysis

The results obtained were statistically analyzed using Graph Pad Prism software, California, U.S.A. Comparisons between all the experimental groups of the study were made using one-way analysis of variance (ANOVA). Results were expressed as mean ± SD. Comparison between multiple groups were analyzed by One Way ANOVA followed by Bonferroni’s multiple comparison and unpaired t-test was used to compare the data between the two groups. Differences were considered statistically significant for *p* < 0.05.

## Results

### Reactive oxygen species accumulation

**Figure 1:**
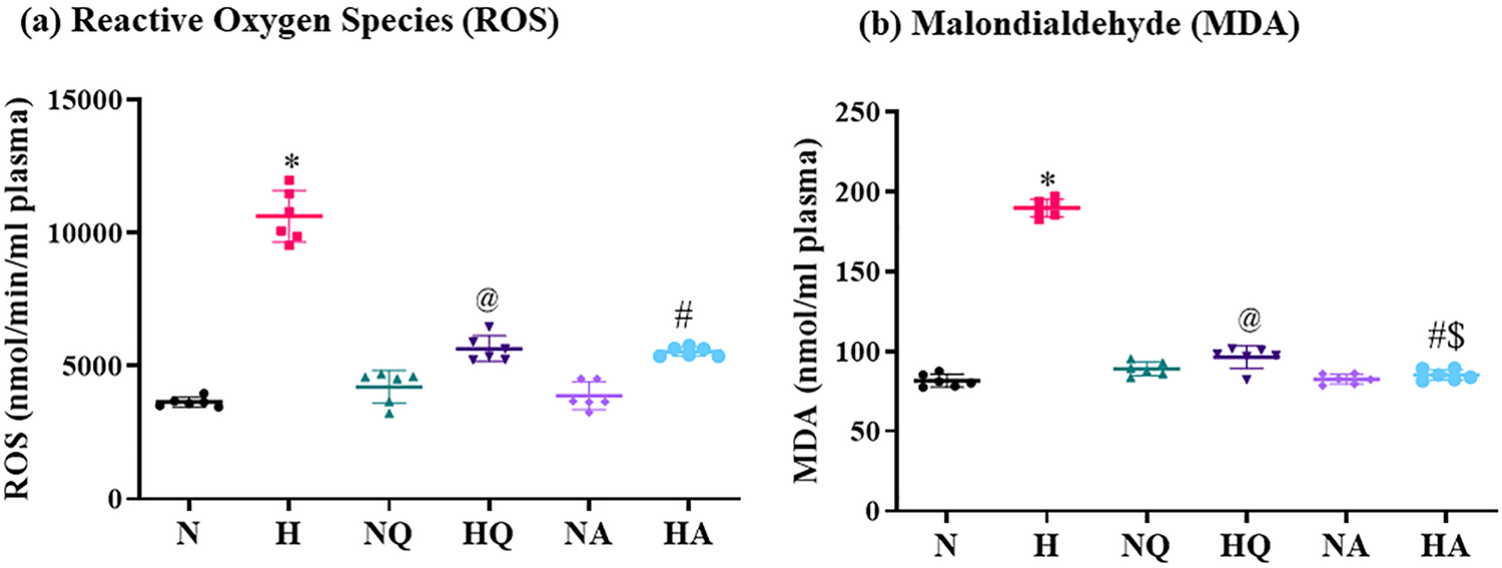
Effect of quercetin and acetazolamide prophylaxis on oxidative stress in plasma of rats exposed to hypobaric hypoxia at 25,000 ft. (7,620 m) for 12 h. (**a**) Reactive Oxygen Species (ROS) *p < 0.001 N vs H, @ *p* < 0.001 H vs HQ, #*p* < 0.001 H vs HA (**b**) Malondialdehyde (MDA) *p < 0.001 N versus H, @*p* < 0.001 H versus HQ, #*p* < 0.001 H versus HA, $*p* < 0.01 HQ versus HA. N = Normoxia control, H = Hypoxia, NQ = Normoxia + Quercetin, HQ = Hypoxia + Quercetin, NA = Normoxia + Acetazolamide, HA = Hypoxia + Acetazolamide.

Reactive oxygen species generated in all the experimental groups is depicted in Fig. 1a. As a consequence of hypoxic stress, a significant increment in reactive oxygen species in plasma was detected in hypoxia exposed group in comparison with normoxia control group (3-fold ↑). However, quercetin and acetazolamide pretreatment 1 h prior to hypoxia exposure significantly (*p* < 0.001) restored the ROS generated in plasma and thus a reduction in its levels was observed (1.3-fold ↓ and 1.8 fold ↓, respectively) as compared with hypoxia exposed group. However, quercetin and acetazolamide treated normoxia control animals exhibited no change in their plasma ROS levels. Both the drugs managed to attenuate the plasma ROS production under hypoxia equally, hence, in comparison, no significant difference was observed in their activity in hypoxia exposed quercetin and acetazolamide pretreated group.

### Malondialdehyde adduct formation

As a result of hypoxia induced excessive ROS production, lipid peroxidation via super-oxides interaction with membrane poly unsaturated fatty acids (PUFA) leads to over production of MDA. This can be evidently noted in Fig. 1b where a significant elevation (*p* < 0.001) in MDA levels in plasma was observed in hypoxia exposed group when compared to normoxia controls (2.5 fold ↑). However, a significant reduction in plasma MDA levels were seen in animal group pretreated with quercetin and acetazolamide as compared to hypoxia exposed group (2-fold ↓ and 2.4-fold ↓, respectively). Drug pretreated both, normoxia groups exhibited no significant change with respect to normoxia control. Administration of acetazolamide reduces the MDA production in plasma of rats by 1.03-fold ↓ when compared with hypoxia exposed quercetin prophylactic group (*p* < 0.01).

### Glutathione peroxidase assessment

**Figure 2:**
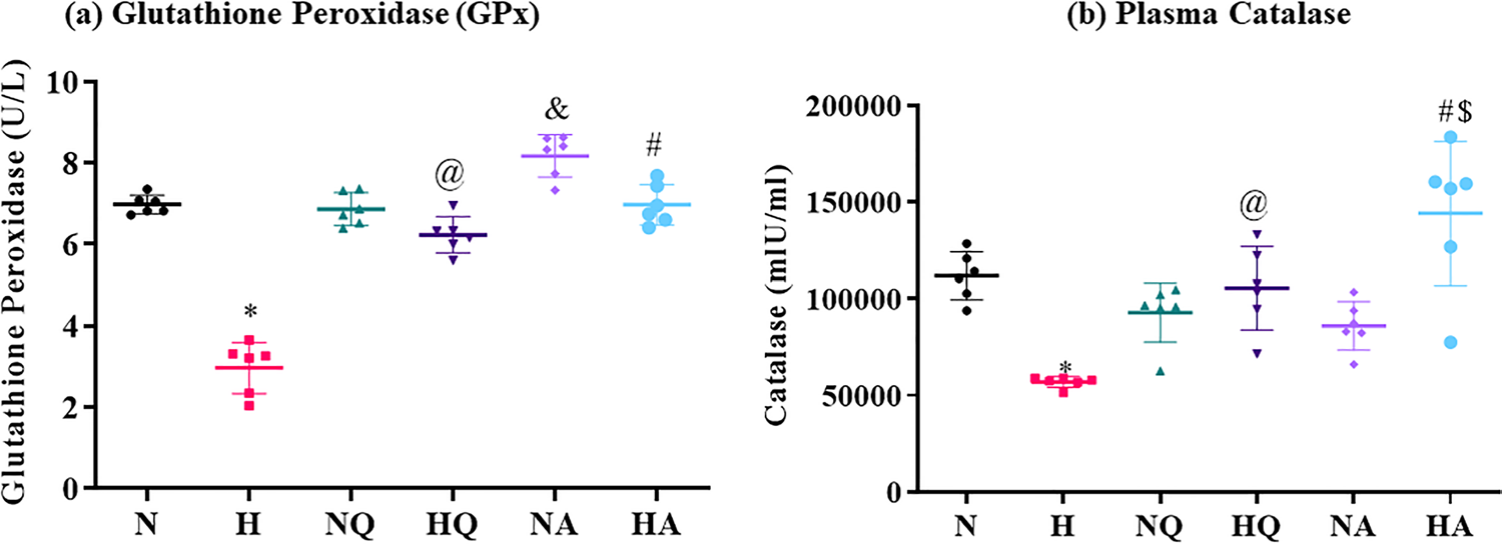
Effect of quercetin and acetazolamide administration on anti-oxidant levels in plasma of rats exposed to hypobaric hypoxia at 25,000 ft. (7,620 m) for 12 h. (**a**) Glutathione Peroxidase (GPx) *p < 0.001 N versus H, @ *p* < 0.001 H versus HQ, #*p* < 0.001 H versus HA, &*p* < 0.01 N versus NA (**b**) Catalase *p < 0.001 N versus H, @*p* < 0.01 H versus HQ, #*p* < 0.001 H versus HA, $*p* < 0.05 HQ versus HA. N = Normoxia control, H = Hypoxia, NQ = Normoxia + Quercetin, HQ = Hypoxia + Quercetin, NA = Normoxia + Acetazolamide, HA = Hypoxia + Acetazolamide.

Under hypobaric hypoxic conditions, antioxidant ability of body, weakens which is apparently, visible in results obtained in the present study, which is depicted in Fig. 2a. Plasma glutathione peroxidase activity (GPx activity) significantly (*p* < 0.001) diminished in hypoxia exposed group as compared to the normoxic group (3.5-fold ↓). Although, quercetin and acetazolamide administered groups prior to hypoxia exposure manifested a significant increment in plasma GPx levels (2-fold ↑ and 2.3-fold ↑, respectively) as compared with hypoxia exposed group. However, quercetin treated normoxia group showed unchanged plasma GPx levels when compared to normoxia control. Both the drugs achieved to enhance plasma GPx levels equally. However, an unusual elevation (1.2-fold ↑) in plasma GPx activity was observed in acetazolamide supplemented normoxia animals as compared to normoxia control (*p* < 0.01).

### Catalase estimation

Similar to the results obtained in plasma GPx, plasma catalase also experienced a significant upsurge (2.4-fold ↑ and 3-fold ↑) in animal group fed with quercetin and acetazolamide, respectively 1 h prior to hypoxia as compared with hypoxia exposed group that depicted a significant downfall (2.8-fold ↓) in plasma GPx levels under hypoxic stress as compared with control group. However, no significant changes were observed in catalase activity of drugs supplemented to normoxia when compared with normoxia control group (Fig. 2b). Higher catalase activity in plasma (1.5-fold↑) of rats was observed in acetazolamide pretreated hypoxic group in comparison with the quercetin treated hypoxia group. This hyperactivity in plasma GPx activity was 1.3-folds ↑ higher than normoxia control.

### Estimation of plasma renin activity

**Figure 3:**
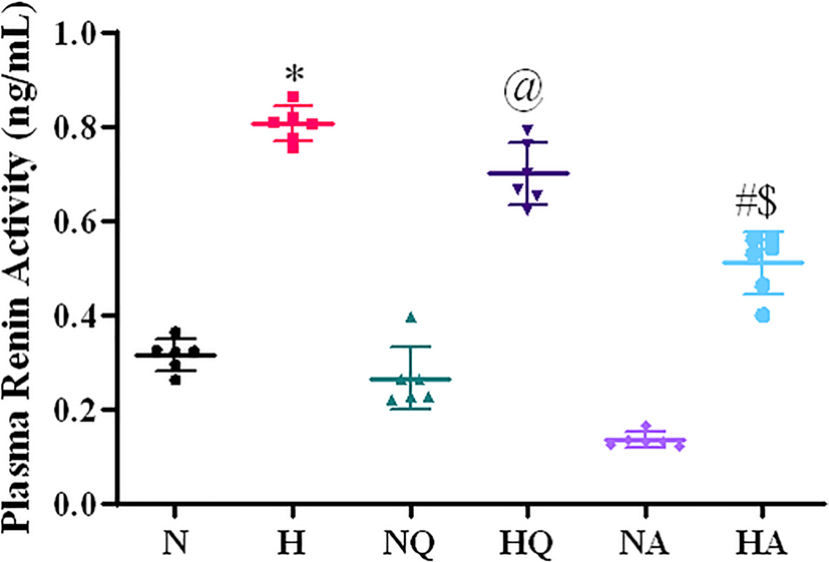
Effect of quercetin and acetazolamide supplementation on Plasma Renin Activity (PRA) of rats exposed to hypobaric hypoxia at 25,000 ft. (7620 m) for 12 h. *p < 0.001 N versus H, @*p* < 0.001 N versus HQ, #*p* < 0.01 H versus HA, $ *p* < 0.05 HQ versus HA. N = Normoxia control, H = Hypoxia, NQ = Normoxia + Quercetin, HQ = Hypoxia + Quercetin, NA = Normoxia + Acetazolamide, HA = Hypoxia + Acetazolamide.

Plasma renin activity showed a significant increment (2.6 fold ↑) under hypoxic stress (*p* < 0.001) when compared with control, however quercetin administration has shown no significant effect on plasma renin activity, whereas acetazolamide supplemented animals showed reduced plasma renin activity significantly (*p* < 0.01) as compared to the hypoxia exposed group. Further, quercetin does not appear to be altering the plasma renin activity in normoxia control group, as no noticeable change was observed. However, supplementation with acetazolamide to normoxia group reduced the plasma renin activity, but results obtained were not significant (Fig. 3). A significant reduction (1.4-fold ↓) in plasma renin activity was observed in acetazolamide pretreated hypoxia group with respect to the quercetin administered hypoxia exposed animals (*p* < 0.05).

### Angiotensin I and II determination

**Figure 4:**
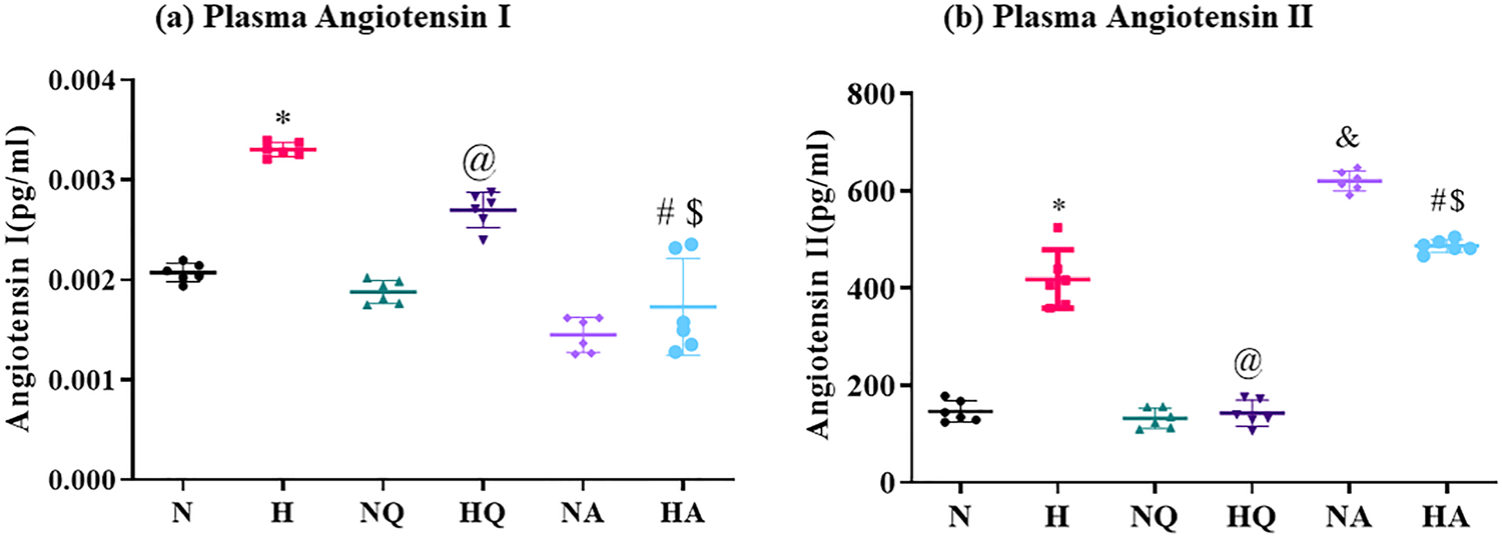
Effect of quercetin and acetazolamide supplementation on (**a**) Angiotensin I levels and (**b**) Angiotensin II levels in plasma of rats exposed to hypobaric hypoxia at 25,000 ft. (7620 m) for 12 h. (**a**) Angiotensin I: *p < 0.001 N versus H, @*p* < 0.01 H versus HQ, #*p* < 0.001 H versus HA, $*p* < 0.001 HQ versus HA (**b**) Angiotensin II: *p < 0.001 N versus H, @*p* < 0.001 H versus HQ, #*p* < 0.05 H versus HA, $*p* < 0.001 HQ versus HA, & *p* < 0.0.01 N versus NA. N = Normoxia control, H = Hypoxia, NQ = Normoxia + Quercetin, HQ = Hypoxia + Quercetin, NA = Normoxia + Acetazolamide, HA = Hypoxia + Acetazolamide.

Hypoxia exposure resulted in  a significant increase in Ang I and II (1.5-fold↑, 3.2-fold↑, respectively) levels as compared to normoxia control. However, both the drugs aided in reduction of Ang I and II (1.25-fold ↓and 1.94-fold ↓, respectively) levels as compared to hypoxia control animals Fig. 4 a, b. However, we have observed that rats receiving acetazolamide both under normoxia and hypobaric hypoxia exposures showed a significant reduction (*p* < 0.001) in plasma angiotensin I compared to normoxia control. In contrast to these results, a significant drastic increment in Ang II levels was observed in acetazolamide normoxia and hypoxia groups when compared with normoxia control group (4-fold ↑ and 2-fold ↑ respectively) (*p* < 0.001) (Fig. 4 a, b).

### Activity of ACE 2

**Figure 5:**
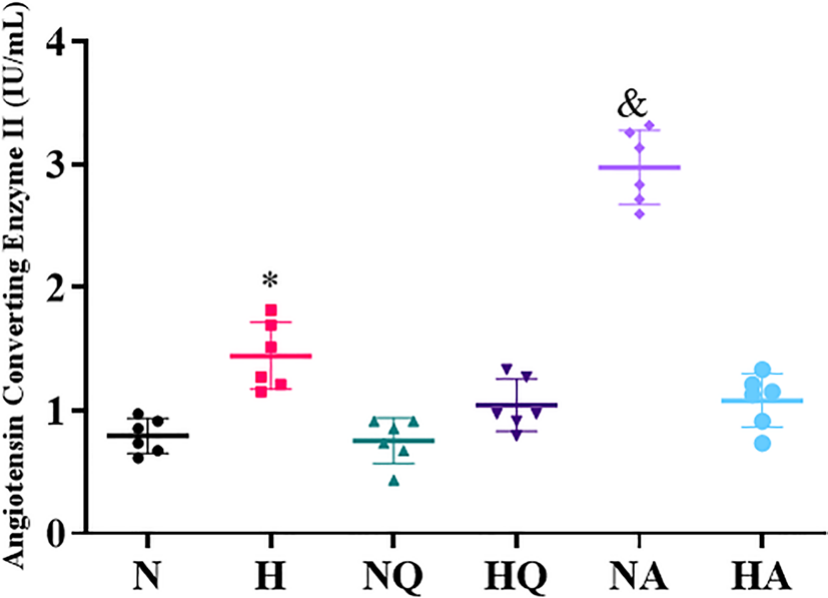
Effect of quercetin and acetazolamide supplementation on Angiotensin Converting Enzyme 2 activity in plasma of rats exposed to hypobaric hypoxia at 25,000 ft. (7620 m) for 12 h. *p < 0.001 N versus H, & *p* < 0.001 N versus NA. N = Normoxia control, H = Hypoxia, NQ = Normoxia + Quercetin, HQ = Hypoxia + Quercetin, NA = Normoxia + Acetazolamide, HA = Hypoxia + Acetazolamide.

Hypoxia exposed group exhibited a significant augmentation (2-fold ↑) (*p* < 0.001) in angiotensin- converting enzyme 2 activity as opposed to control (Fig. 5). Whereas, rats receiving quercetin and acetazolamide prior to hypoxia exposure exhibited absolutely no ACE 2 enzyme activity as compared with hypoxia exposed group which is more or less same as that of normoxia rats. Although, no noticeable change was observed in quercetin fed normoxia group as compared with normoxia control, but acetazolamide fed normoxic group exhibit abnormally enhanced levels of ACE 2 enzyme activity (3-fold ↑).

### Aldosterone estimation in plasma and urine

**Figure 6:**
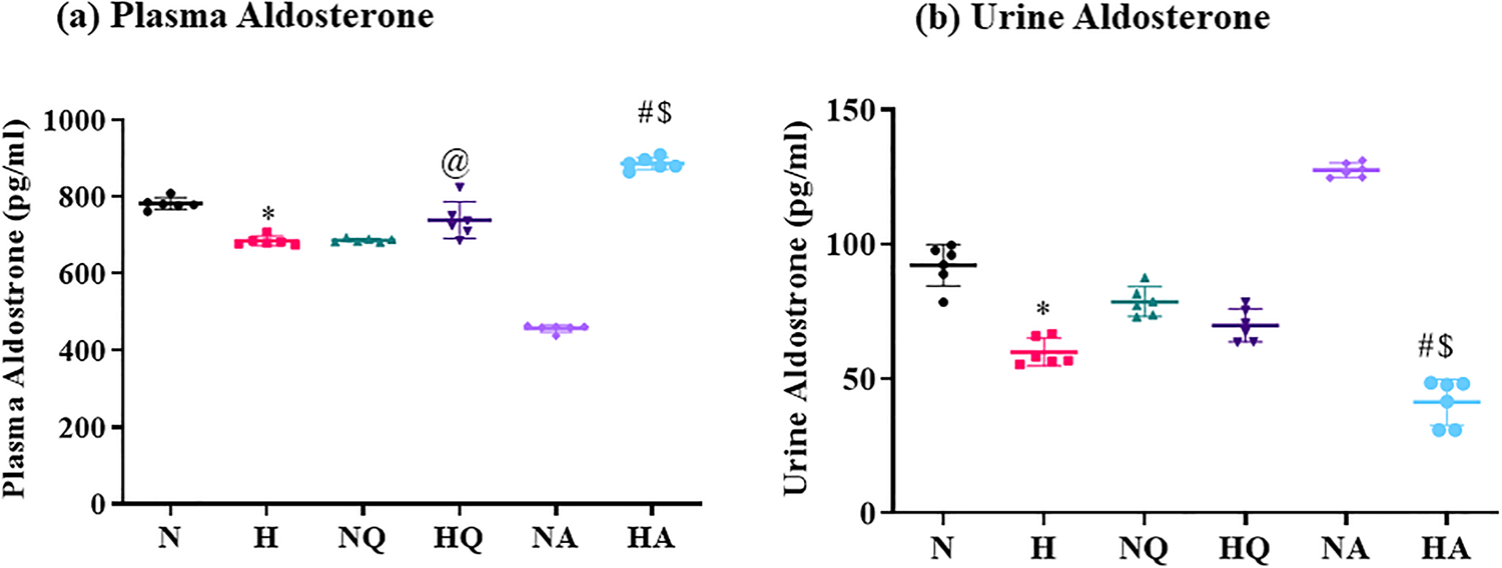
Effect of quercetin and acetazolamide supplementation on aldosterone levels in (**a**) plasma (*p < 0.001 N vs. H, @*p* < 0.01 H vs. HQ, #*p* < 0.001 H vs. HA, $*p* < 0.001 HQ vs. HA) (**b**) urine (*p < 0.001 N vs. H, #*p* < 0.001 H vs. HA, $*p* < 0.001 HQ vs. HA) of rats exposed to hypobaric hypoxia at 25,000 ft. (7620 m) for 12 h. N = Normoxia control, H = Hypoxia, NQ = Normoxia + Quercetin, HQ = Hypoxia + Quercetin, NA = Normoxia + Acetazolamide, HA = Hypoxia + Acetazolamide.

Release of aldosterone by adrenal gland in blood stream is initiated by Angiotensin II. A major downfall in plasma aldosterone levels were observed in plasma and urine of hypoxia exposed groups with respect to normoxia control group as depicted in Fig. 6a, b. Hypoxia exposed animals exhibited a significant (1.1-fold ↓, 1.5 fold ↓, respectively) reduction in levels of aldosterone in plasma and urine as compared to control group. Administration of quercetin and acetazolamide, significantly (*p* < 0.01) increased the aldosterone levels in plasma. Quercetin fed normoxia group exhibited no significant change in plasma aldosterone levels as compared to normoxia control. However, acetazolamide prophylaxis reduced the aldosterone levels in normoxia rats significantly with respect to normoxia control. Additionally, our results manifested an unusual elevation of aldosterone levels in plasma of acetazolamide pretreated hypoxia exposed groups which were found not only in comparison with normoxia control group but also found to be enhanced in comparison to hypoxia and hypoxia exposed quercetin fed groups (1.1-fold ↑) (*p* < 0.001) (Fig. 6a).

Quercetin supplemented hypoxia exposed group exhibited no significant change in aldosterone levels in urine when compared with hypoxia exposed group. However, administration of acetazolamide decreased the urine aldosterone levels under hypoxia stress with respect to hypoxia exposed group. However, urine aldosterone levels exhibited an abnormal upsurge in acetazolamide fed normoxia group as compared to normoxia control (Fig. 6b). Rats prior treated with acetazolamide and exposed to hypoxia showed a significant reduction in the aldosterone levels (1.8-fold↓) in urine when compared with quercetin prophylactic hypoxic group of rats (*p* < 0.001).

### Protein expression

**Figure 7:**
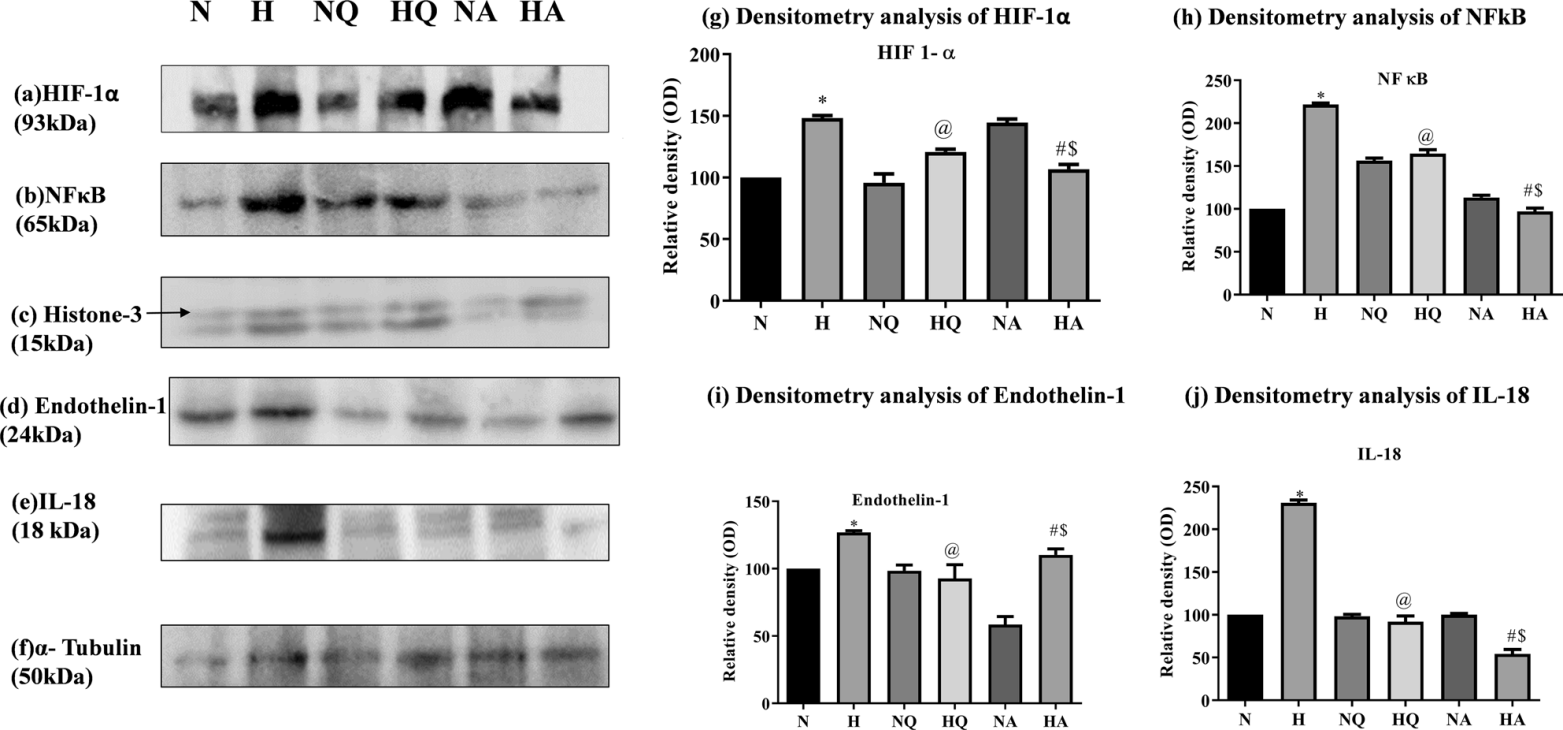
Effect of quercetin and acetazolamide supplementation on protein expressions of (**a**) Hypoxia-Inducible Factor 1-alpha (HIF-1α) (**b**) Nuclear Factor kappa B (NFκB) (**c**) Histone-3 (H-3) (**d**) Endothelin-1 (**e**) Interleukin-18 (IL-18) and (**f**) α-Tubulin in kidneys of rats exposed to hypobaric hypoxia at 25,000 ft. (7620 m) for 12 h. (**g**) Densitometry analysis of HIF -1α (**h**) NFκB (**i**) Endothelin-1 (**j**) IL-18. *p < 0.001 N versus H, @*p* < 0.001 H versus HQ, #*p* < 0.001 H versus HA, $*p* < 0.001 HQ versus HA. N = Normoxia control, H = Hypoxia, NQ = Normoxia + Quercetin, HQ = Hypoxia + Quercetin, NA = Normoxia + Acetazolamide, HA = Hypoxia + Acetazolamide. (All Original blots are available in Supplementary Figure S1).

Hypobaric hypoxia induced inflammation and vasoconstriction in kidney tissues of normoxia and hypoxia exposed rats were analyzed for the protein expression studies of transcriptional factors (HIF-1α, NFκB) and the genes regulated by them (ET-1 and IL-18 respectively; Fig. 7a, b, d and e). The hypoxia exposure resulted in upregulation of HIF-1α and NFκB significantly (*p* < 0.001) in comparison with the normoxia control; and so is the case with their representative proteins i.e. ET-1 and IL-18 respectively. Preconditioning with quercetin in normoxia group does not lead to any alterations in any of these transcriptional factors and their associated proteins in comparison with normoxia control group. However, pretreatment with acetazolamide under normal conditions showed a significant elevation in the expression of HIF-1α in kidneys of rats. Quercetin aids in stabilization of HIF-1α expression along with reduced expression of proteins NFκB, IL-18 and ET-1 in kidney tissues of hypoxia exposed groups, i.e. lowering them to normal values. However, acetazolamide administration reduces their expressions far lower than normal values when compared with normoxia control. Protein expressions of ET-1 rises significantly under hypoxic stress when compared with normoxia control group. Both the drugs managed to reduce the ET-1 protein expression in kidneys, significantly when compared with hypoxia exposed group (*p* < 0.001 and *p* < 0.05, respectively). However, acetazolamide appears to be less efficient than quercetin as the reduction attained by quercetin was far better than acetazolamide (*p* < 0.05). The densitometry analysis of the HIF-1α, NFκB, ET-1 and IL-18 protein expressions in kidneys of rats have been represented in Fig. 7g, h, i and j respectively.

#### Binding of quercetin and acetazolamide with ACE 2

**Figure 8:**
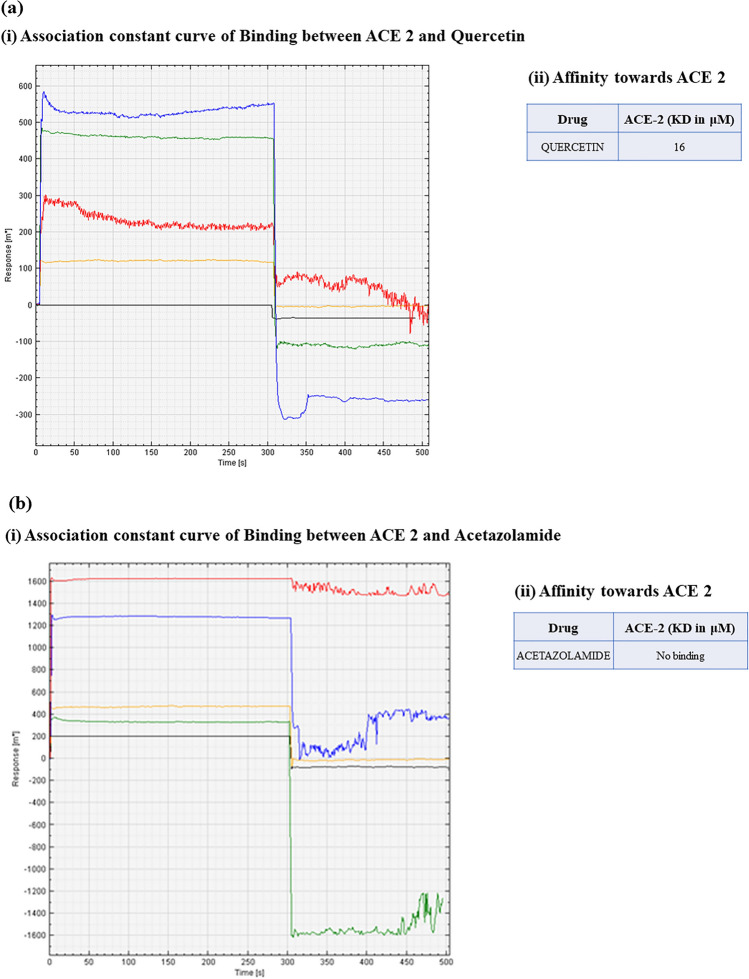
Binding potential of (**a**) Quercetin with Angiotensin Converting Enzyme 2 (ACE 2) represented in terms of (i) Association constant curves and (ii) Dissociation constant (KD) values and (**b**) Acetazolamide with ACE 2 depicted in terms of (i) Association constant curve and (ii) Dissociation constant (KD) values that shows no binding of acetazolamide with ACE 2.

The association kinetics of the quercetin using a concentration gradient ranging from 1.5 to 100 µM against ACE 2 exhibited a sigmoidal pattern of association curve by using Surface Plasmon Resonance Spectroscopy (SPR). SPR was performed as represented in Fig. 8a, b. Binding affinity of quercetin and acetazolamide against ACE 2 was expressed in terms of Dissociation Constant (KD) value, which is considered to be inverse of affinity constant (binding affinity α 1/KD). Based on the Michaelis–Menten’s equation (KD is inversely proportional to affinity), quercetin binds with ACE 2 with KD value of 16 μM (Fig. 8a, i and ii). Whereas, no such binding was observed when ligand ACE 2 was run with analyte acetazolamide (Fig. 8b, i and ii,).

#### Ion analysis

The data obtained in the present study, does not depict a significant change in levels on Na^+^ and K^+^ ions under hypobaric hypoxia, however, slight reduction in levels of both of these ions was observed. Similarly, no alteration was observed in quercetin and acetazolamide supplemented normoxic and hypoxic groups. Further, Cl^−^ ions seen to be significantly decreased in hypoxia exposed groups as compared with normoxia control (*p* < 0.001). Administration of quercetin and acetazolamide aids in elevation (*p* < 0.001 and *p* < 0.01, respectively) of Cl^−^ ions when compared with hypoxia control. A significant increment in hemoglobin levels were also observed in hypoxia exposed group when compared with normoxia control. Quercetin administration raised Hb levels in hypoxia exposed group when compared with hypoxia control, however, this increment was not significant in comparison to hypoxia exposed rats. Similarly, Acetazolamide prophylaxis also could not be able to increase the Hb levels over hypoxia exposed animals and hence, results were non-significant (Table 1).

**Table 1:** Effect of quercetin and acetazolamide administration on ion analysis in plasma of rats exposed to hypobaric hypoxia at 25,000 ft. (7620 m) for 12 h. (**A**) Sodium (Na^+^), (**B**) Potassium (K^+^), (**C**) Chloride (Cl^-^) (*p < 0.001 N vs. H, @*p* < 0.001 H vs HQ, #*p* < 0.01 H vs. HA, $*p* < 0.001 HQ vs. HA) and (**D**) Haemoglobin (Hb) (*p < 0.05 N vs. H).

S. No	Parameters	N	H	NQ	HQ	NA	HA
A	Sodium (Na^+^) (mmol/L)	140 ± 3.2	136.8 ± 1.47	138.8 ± 1.9	137.6 ± 0.47	137.5 ± 1.8	141.6 ± 1.37
B	Potassium(K^+^) (mmol/L)	4.8 ± 0.6	4.25 ± 0.1	4.68 ± 0.17	4.5 ± 0.11	4.3 ± 0.16	4.5 ± 0.19
C	Chloride (Cl^-^) (mmol/L)	100 ± 1.4	86.16 ± 2.2*	101.5 ± 1.8	101 ± 1.4^@^	113.8 ± 2.2	88.3 ± 2.6^#$^
D	Haemoglobin (Hb) (mg/dl)	12.8 ± 0.5	14.8 ± 0.3*	13.5 ± 0.4	15.3 ± 0.7	11.6 ± 0.4	14.1 ± 0.8

Ion concentrations are expressed in mmol/L whereas Hb is in mg/dl.

N = Normoxia control, H = Hypoxia, NQ = Normoxia + Quercetin, HQ = Hypoxia + Quercetin, NA = Normoxia + Acetazolamide, HA = Hypoxia + Acetazolamide.

## Discussion

This study was conducted to analyze the role of Renin–angiotensin–aldosterone pathway and its effect on kidneys of rats under hypobaric hypoxic stress. Further, the aim of this study was also, to find out, how this pathway can be targeted to facilitate the rapid ascent to high altitude by sojourners and visitors. Hence, we first, studied the effect of hypoxia on various targets of RAAS pathway, and then -we have analyzed if quercetin prophylaxis is able to ameliorate the alterations caused by hypobaric hypoxia. The results obtained suggest that quercetin was able to regulate the components of this pathway in appropriate direction depending on the requirements via mitigating the excessive renal oxidative stress and inflammation. Finally, we have tried to compare the benefits attained by quercetin prophylaxis with acetazolamide (Diamox) a well-known drug recommended for enhancing the acclimatization process while ascending to high altitudes.

The present work demonstrates the nephro-protective activity of quercetin in rats exposed to acute hypobaric hypoxia. In this study, quercetin was able to prevent the oxidative damage induced in kidneys due to hypoxic stress measured in terms of reactive oxygen species generated. In accordance with previous literature, the study presented here observed a significant imbalance in redox system. Nonetheless, the extreme ROS production was quenched by quercetin, lipid peroxidation decreased, consequently. Similar outcomes were obtained in a study conducted by Pietruck et al. that demonstrated a protective effect of quercetin on freshly isolated renal proximal tubules by inhibition of lipid peroxidation^33^. They have reported that, quercetin was able to inhibit the hypoxia induced functional and structural injuries in renal tubules, but did not show any effect on tubular energy metabolism. The authors of this study further reported that LDH (lactate dehydrogenase)—a marker for tissue injury was released after 15 min of hypoxia exposure was found to be completely reduced by quercetin treatment^33^.

Moreover, GPx and catalase that enzymatically catabolizes ROS^34^ experienced a significant downfall under hypoxic stress. Although, quercetin being an antioxidant itself enhanced their activity upon administration, prior to hypoxia exposure. However, their (GPx and catalase) abnormally elevated levels in acetazolamide pretreated hypoxia exposed group compared to control group, suggested that, acetazolamide administration leads to excessive peroxidase activity which is implicated in oxidative damage of cells and tissues, thus contributes to ongoing maladies of high altitudes. The excess production of GPx and catalase levels also exert deleterious effects such as activation of cell death, damage to macromolecule etc. Reports also indicates increased GPx expression in some cancer cells that may aid in tumor progression and various disorders^35–37^. Hence, the study infers that these (GPx and catalase) antioxidants play dichotomous role under hypoxic stress. So antioxidant supplements or drugs like acetazolamide should be used with caution.

Previously conducted high altitude studies suggests that renal excretory response is in a constant state of change under decreased partial pressure of atmospheric oxygen, continuously attempting to maintain plasma sodium and electrolyte balance^38^. This study witnessed no significant change in hypoxia exposed group in plasma Na^+^ and K^+^ ion levels however, Cl^−^ ion levels exhibited a significant fall when compared with normoxia control. Further, pretreatment with quercetin and acetazolamide helps in restoration of Cl^−^ ions efficiently when compared with hypoxia exposed group. No discrete relationship has been found between sodium excretion and extent of exposure to high altitude. Several studies reported a decrease in plasma Na^+^ ion levels with no change in K^+^ and Cl^−^ ions^24,38^. Whereas, Khan et al. observed that, variable sodium excretion rate from higher levels to no change at all in acclimatizing subjects^39,40^. The present study also showed that, diminished chloride levels leads to respiratory alkalosis at high altitude that might deteriorates kidney and give rise to fatal conditions^41^. Thus, quercetin and acetazolamide supplementation significantly restore Cl^−^ ions. However, quercetin manages to elevate Cl^−^ ions almost to normoxia control and acetazolamide fails to do so indicating towards the efficacy of quercetin over acetazolamide.

Quercetin plays a cardio-renal protective role which is achieved by its anti-oxidant and anti- inflammatory potential thus acting in an immunomodulatory manner^42,43^. The data generated here, indicates a plausible therapeutic effect of quercetin on renin–angiotensin–aldosterone axis that showed a protective effect^44^. A study by Xuan et al. illustrates the improvement in tubulointerstitial kidney damage achieved by polarization of M1/M2 macrophages along with reduction in iNOS (Inducible Nitric Oxide Synthase) and IL-12^45^. This study indicates that the events involved in achieving this pharmacological mechanism includes protein translation as the effects of quercetin that were associated with negatively regulated activities of NF-κB^45^.

A study evaluating nephroprotective activity of quercetin against I/R and inhibition of RAAS pathway, performed a comparison between Lisinopril, losartan and quercetin and found that Lisinopril was a potent ACE inhibitor however, does not present protective effects against I/R-induced Acute Kidney Injury. Whereas, Losartan which is an important AT1-R blocker (ARB), exhibited moderate effects in comparison with quercetin^46^. Moreover, AT-1 inhibition results in surplus ACE that leads to the activation of Angiotensin-(1-7) pathway mediated by ACE2 imparting anti-inflammatory and antioxidant effects^47^.

The present study showed an insignificant reduction in plasma renin activity in quercetin supplemented hypoxia exposed animals as compared to hypoxia exposed group. Whereas, administration of acetazolamide reduced the renin activity significantly with respect to hypoxia exposed group. Furthermore, a significant reduction in Ang I was achieved by supplementation of both the drugs. In continuation to that acetazolamide failed to reduce the Ang II under hypoxic conditions whereas quercetin significantly facilitated the reduction in Ang II levels. This indicate towards the antihypertensive activity of quercetin over acetazolamide. Apart from this, an abnormal elevation in Ang II levels were observed in acetazolamide supplemented normoxia group with respect to normoxia control groups that suggests adverse effects of acetazolamide in normal conditions. A study by Litchblau et al., 2021 witnessed a slight metabolic acidosis in hypoxic conditions on injecting acetazolamide however, no significant modifications of PaCO_2_ and PaO_2_ were observed. Hence, occurrence of renal carbonic anhydrase inhibition on ventilation and blood gases were not speculated in this study^48^. Moreover, most commonly reported adverse effects of acetazolamide ingestion includes paresthesia, dysgeusia and diuresis. It has been hypothesized that some of these side effects may be related to the extent of metabolic acidosis caused by acetazolamide (via renal bicarbonate excretion which attains a steady state within 1–2 days)^49,50^. Some of these typical acetazolamide related side–effects (nausea, vomiting, confusion and drowsiness) cannot be clearly separated from the symptoms of AMS and might obscured the symptoms of AMS in acetazolamide administered individuals^51^. All the above mentioned findings implies that quercetin maintains the plasma renin activity under hypoxic conditions and facilitates the acclimatization mechanism by efficiently reducing Ang I and Ang II whereas acetazolamide functions by reducing plasma renin activity but fails to maintain the Ang II levels that might lead to damaging effect of hypertension.

A crucial but contradictory molecule of RAAS pathway is ACE 2 that was observed to be inflated under hypobaric hypoxia. ACE 2 is resistant to ACE inhibitors, thus contributing in angiotensin II antagonism^20,52^. The dilemma of whether ACE 2 is involved in high altitude acclimatization through RAAS pathway is extremely crucial, as proximal tubules of kidneys contain the highest density of ACE2 and these peptides (ACE and ACE 2) exhibit different roles within the kidney^53^. RAAS is known to be a pro-inflammatory system and ACE 2 functions as anti-inflammatory molecule by feed-back mechanism. Quercetin and acetazolamide treated animals does not exhibit any alterations in ACE 2 levels under hypobaric hypoxia. Stabilization of HIF-1α lead to maintain the oxidative homeostasis in quercetin prophylaxis animals better than acetazolamide supplemented animals. Because the acetazolamide receiving rats exhibited a delirious increase in anti-oxidant enzymes both under normoxia and hypoxia (plasma GPx) and under hypoxia (plasma catalase). Nevertheless, attenuation of upregulated NF-κB followed by down regulation of IL-18 compared to hypoxia control indicates that these two drugs helps in diminishing the inflammation under hypoxic stress. However, an important observation noted in this study was that, the reduction of IL-18 protein expression by acetazolamide prophylaxis under hypobaric hypoxia was far less than that of normal control. It is speculated that the global inhibition of NF-κB is detrimental and some amount of expression of NF-κB is always required to maintain the immunological homeostasis. In addition to that acetazolamide was found to be less efficient in reducing ET-1 protein expression when compared with quercetin pretreated hypoxia exposed group. Thus, quercetin prevents narrowing of blood vessels better than acetazolamide and thus helps in maintaining the blood pressure.

**Figure 9:**
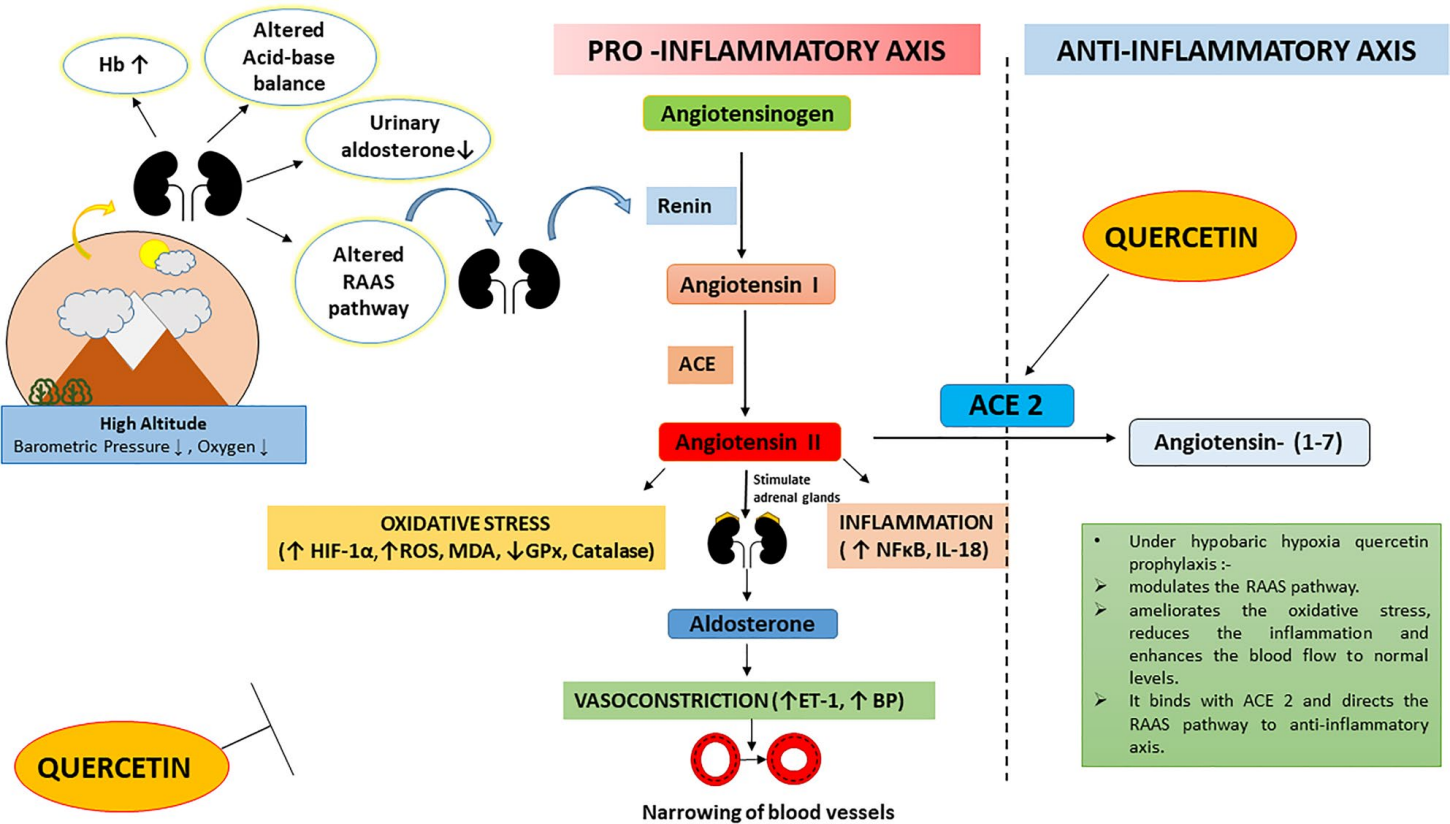
Schematic representation of effect of hypobaric hypoxia on Renin–angiotensin–aldosterone system and plausible mechanism of kidney-protection by quercetin administration in rats. Hb- Hemoglobin, ROS- Reactive Oxygen Species, MDA- Malondialdehyde, GPx- Glutathione Peroxidase, HIF-1α-Hypoxia-Inducible Factor-1α, NFκB-Nuclear Factor kappa B, IL-18- Interleukin-18, ET-1- Endothelin 1, BP- Blood Pressure, RAAS-Renin Angiotensin Aldosterone System, ACE- Angiotensin Converting Enzyme, ACE 2- Angiotensin Converting Enzyme 2, SPR- Surface Plasmon Resonance, ↑-Upregulation, ↓ Down regulation, Ʇ—Attenuation.

We have further tried to figure out the molecular mechanism behind the results obtained in the present study by performing the binding studies (using Surface Plasma Resonance Spectrometry for protein–protein interaction) of quercetin and acetazolamide with anti-inflammatory molecule of this (RAAS) axis i.e. ACE-2. ACE 2 is a transmembrane protein that contains an extracellular zinc binding domain and located at multiple sites like renal tubular epithelium, lung alveolar epithelial cells, arterial and venous endothelial cells etc^20,54,55^. It exclusively cleaves a single C-terminal residue from Ang-II generating Ang-(1-7), thus counterbalances the accumulation of Ang-II formed by the action of ACE. Our data obtained from SPR spectrometry, showed that, a significant strong binding between quercetin and ACE 2 (KD = 16 μM, Fig. 8a, ii) indicating the role of quercetin in modulating the RAAS pathway via ACE 2. No such binding was observed between acetazolamide and ACE 2 (Fig. 8a, b). Therefore, it seems reasonable to infer that, quercetin prophylaxis protects kidneys by modulating the renin activity thereby regulate the renin–angiotensin–aldosterone axis in rats under high altitude hypobaric hypoxic stress, which is schematically illustrated in Fig. 9.

Apart from a being a remarkable antioxidant, Quercetin has also been reported to impart various other biological effects including anti-inflammatory, anti-platelet, anti-viral, cardio and neuro- protection, anti-bacterial and anti-fungal properties etc^56^. It minimizes the risk of metabolic disorders, cardio vascular diseases and certain types of cancer by its proficient ability to restrain free radicals. Studies suggest that Quercetin could prevent various chronic diseases such as atherosclerosis and thrombosis through its anti-platelet and anti-inflammatory nature^57^. Previous studies have revealed that Quercetin inhibit platelet activation by reducing the formation of platelet aggregates and enhancing the amount of morphologically unmodified platelets^58^. This might be due to quercetin's ability to inhibit cyclooxygenase (COX) enzyme that is responsible for platelet activation^59^ on the other side, quercetin can promote the platelet activation inhibitory pathway by binding with glycoprotein IIb/IIIa (GP IIb/IIIa)^58^. Literature has revealed that quercetin can induce apoptosis and cell cycle arrest in different cell lines such as breast, colon, lung and prostate cancers through the modulation of Pl3K/Akt/Mtor and mapk/erk1/2 pathways^60^ on dose dependent manner. This flavonoid has also demonstrated strong bacteriostatic action against numerous disease causing bacteria such as *Helicobacter pylori*, *Staphylococcus epidermidis*, *Campylobacter jejuni* and *Pseudomonas aeruginosa* etc^61^. Quercetin facilitates in reducing the inflammation by impeding the expression of several pro-inflammatory cytokines like IL-18, TNF-α and NFκB^60^. Recently, prophylactic and therapeutic treatment with Quercetin to SARS-CoV 2 patients displayed the anti-viral efficacy of this phyto-flavonoid. It proficiently inhibits several stages of the COVID-19 viral cycles^62,63^. In human cells, Quercetin was found to alter 30% of the genes encoding protein targets of SARS-CoV2, hence possibly interfering with the activities of 80% of these proteins^63^. PLpro, 3CLpro or Mpro, RNA- dependent RNA polymerase (RdRp) and viral spike glycoproteins are some of the viral proteins that were reported to be inhibited by Quercetin^64,65^. Further, quercetin possess immune-modulatory functions and thus it is capable of inhibiting the ‘cytokine storm’ which is a characteristic feature of many viral infections and also main reason to cause morbidity and mortality in severe cases of SARS-CoV2 infection^66^. In the present study also quercetin protected the kidneys from deleterious effects of inflammation due to altered RAAS pathway in rats under hypobaric hypoxia. It is inferred that quercetin having multiple benefits offers an excellent moiety to prevent high altitude illness encountered by soldiers, trekkers, sports persons, mountaineers or sojourns or may be business people and pilgrims vising to high altitude areas. All these findings clearly evidence the potential use of quercetin in clinical applications.

## Conclusion

The study presented here indicates that, the quercetin plays a reno-protective effect under hypobaric hypoxia by neutralizing oxidative stress, augmenting antioxidant system and modulating various components of RAAS pathway in rats. Quercetin binding with ACE 2 attenuates the pro-inflammatory molecules and shifts the pathway to anti-inflammatory axis and thus exhibit protective effects. However, studies targeting specific components of this pathway are required to validate the involvement of this plant flavonoid in different aspects of RAAS.

### Supplementary Information

Supplementary Figure S1.

## Data Availability

All datasets supporting the conclusions of this article are included within the article.
