# Supplementary material for: Quercetin prophylaxis protects the kidneys by modulating the renin–angiotensin–aldosterone axis under acute hypobaric hypoxic stress
Source: Sci Rep. 2024 Mar 31;14:7617. doi: 10.1038/s41598-024-58134-3 (PMC10982295; doi:10.1038/s41598-024-58134-3)

Supplementary File

Title: Quercetin prophylaxis protects the kidneys by modulating the renin-angiotensin-aldosterone axis under acute hypobaric hypoxic stress

Authors: Vaishnavi Rathi[a], Sarada SK Sagi [a*], Amit Kumar Yadav [b], Manoj Kumar [b], Rajeev Varshney [a]
[a] *Defence Institute of Physiology and Allied Sciences, DRDO, Delhi*
[b] *Department of Biophysics, All India Institute of Medical Science, Delhi*

Figure 7

(a) Hypoxia Inducible Factor -1 alpha (HIF-1 α)

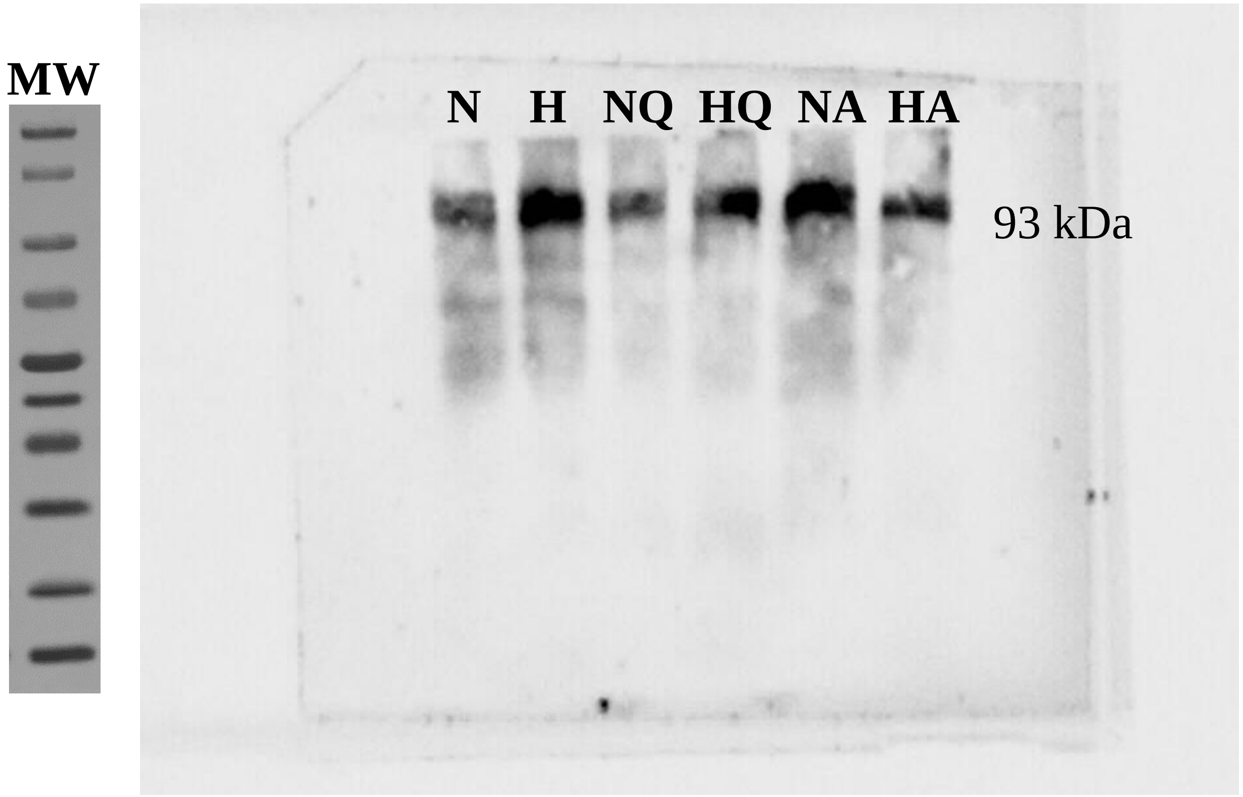

(b) Nuclear Factor kappa B (NF κB)

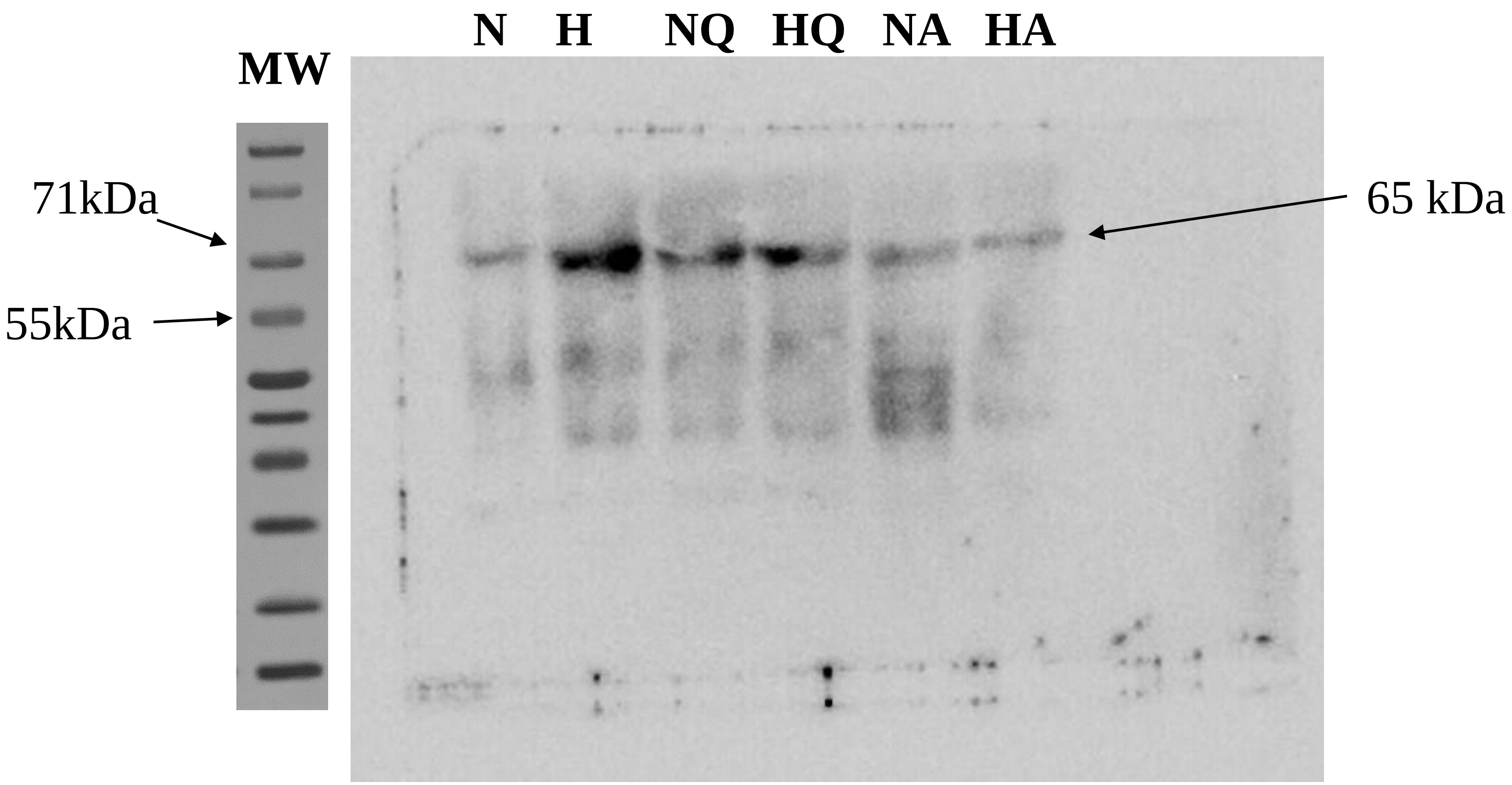

Figure 7

(c) Histone-3

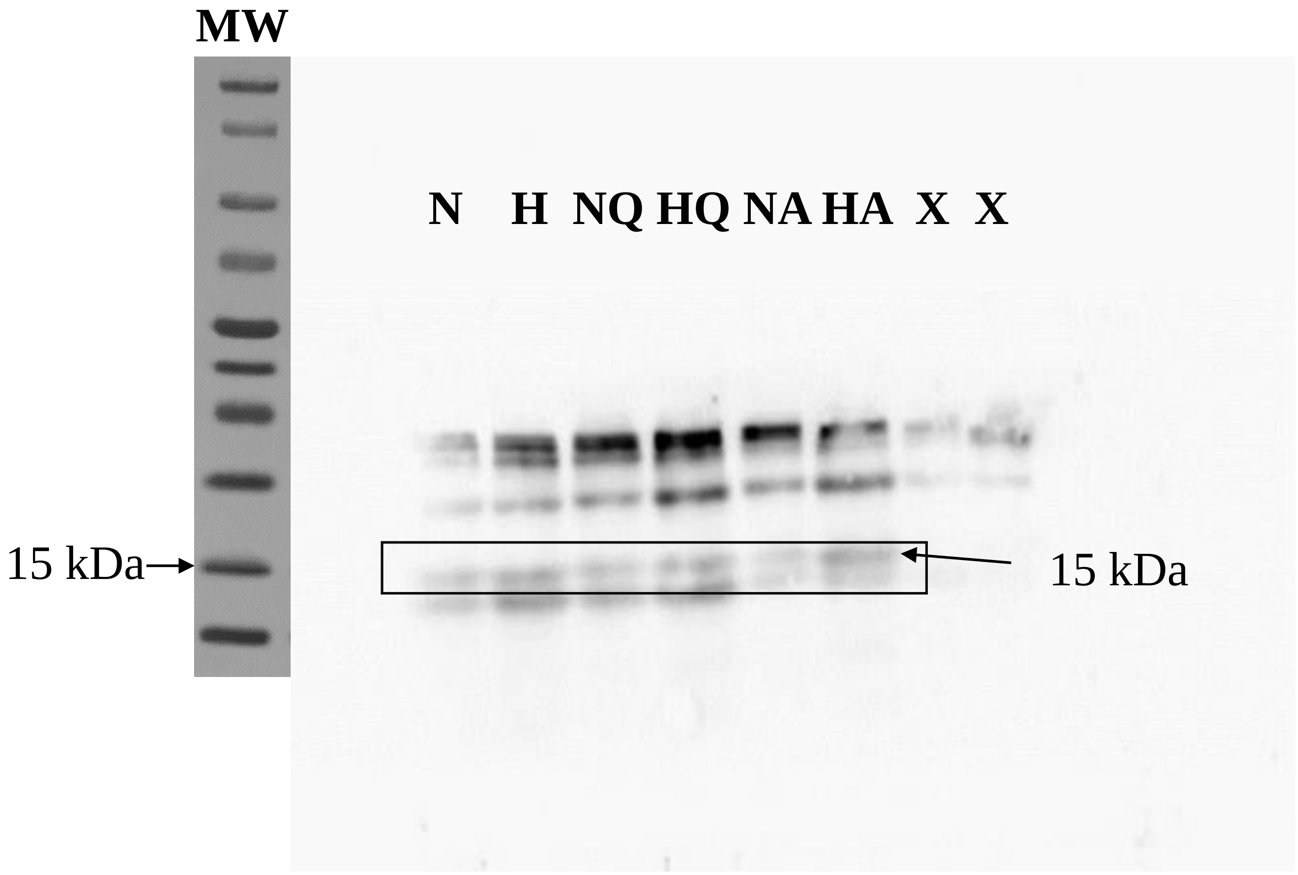

(c) Histone-3

(i) Time of exposure: 5 s

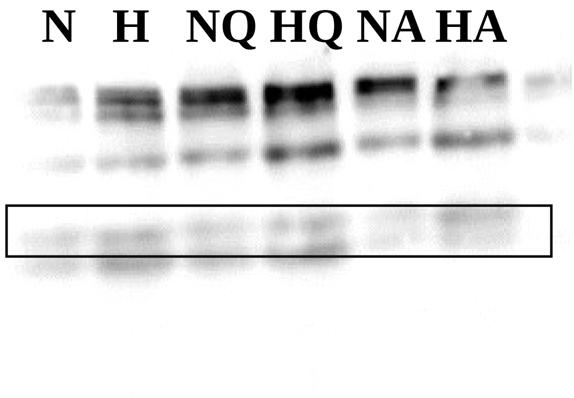

(ii) Time of exposure: 10 s

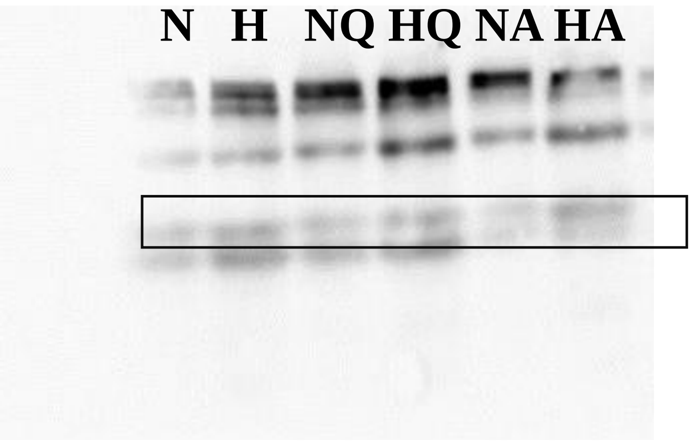

(iii) Time of exposure: 15 s

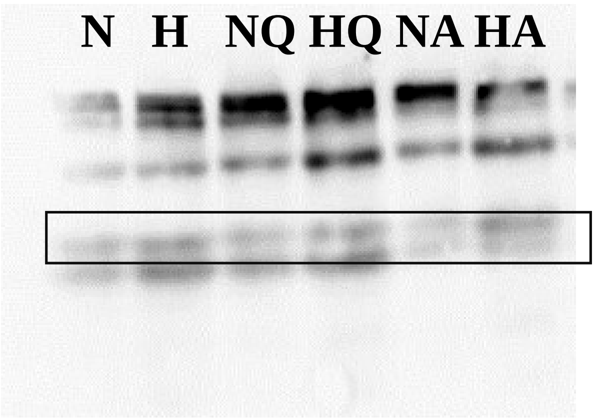

(iv) Time of exposure: 20 s

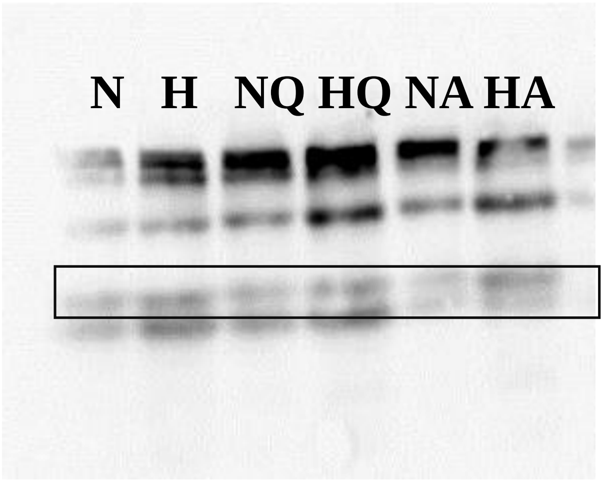

(v) Time of exposure: 30 s

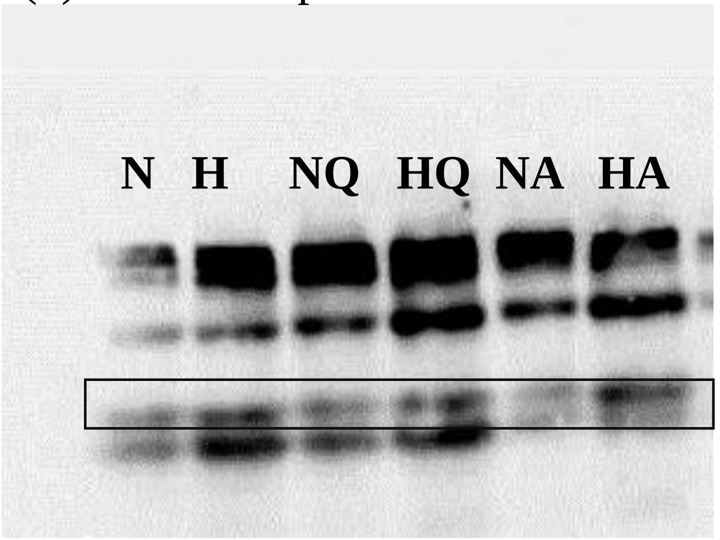

Figure 7

(d) Endothelin-1

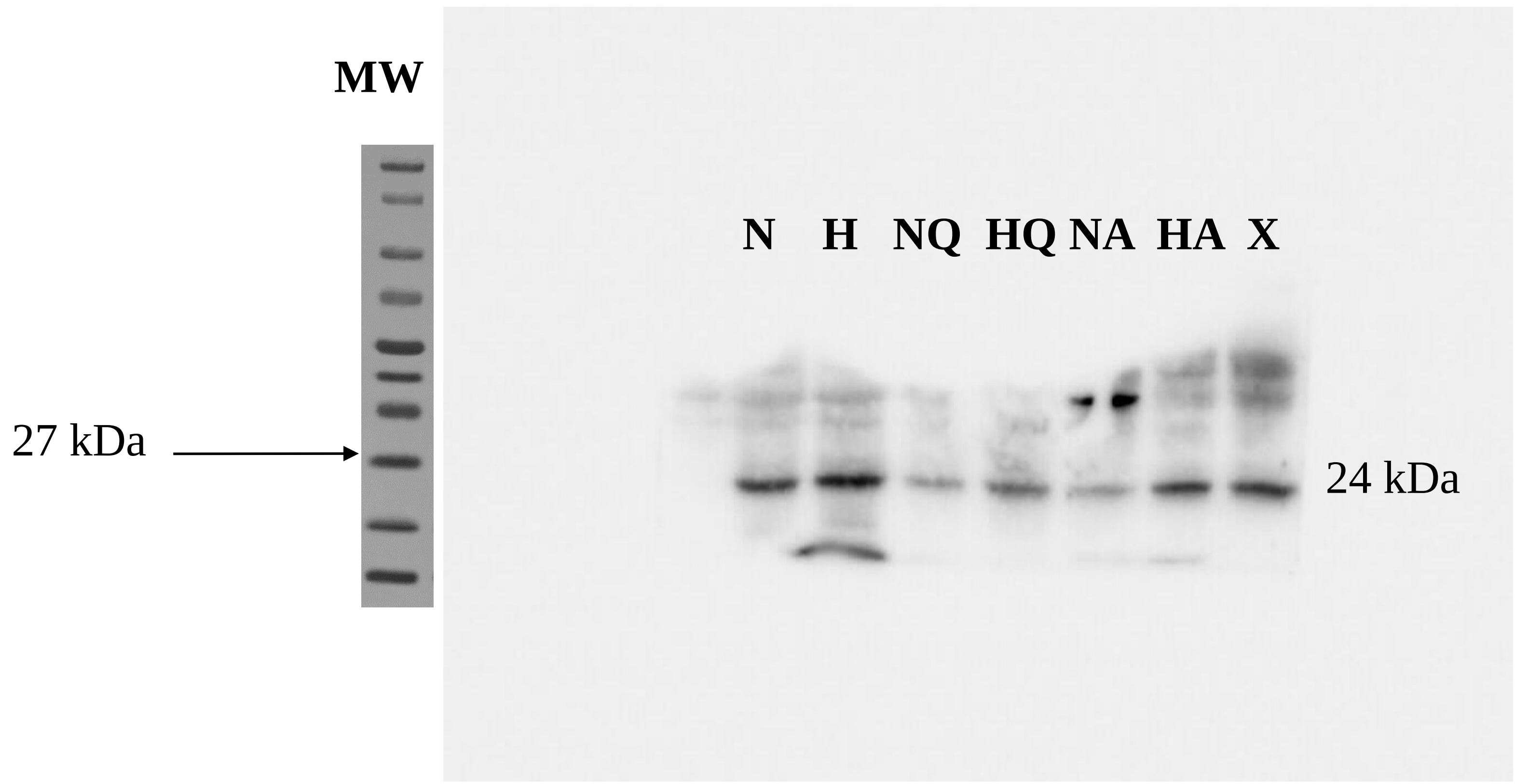

(d) Endothelin-1

(i) Time of exposure: 5 s

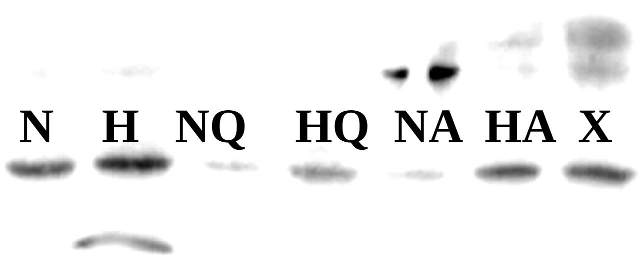

(ii) Time of exposure: 10 s

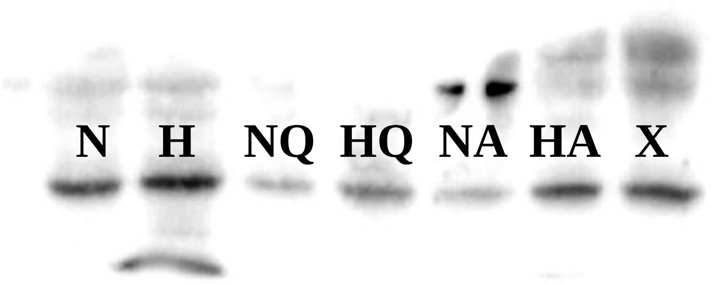

(iii) Time of exposure: 15 s

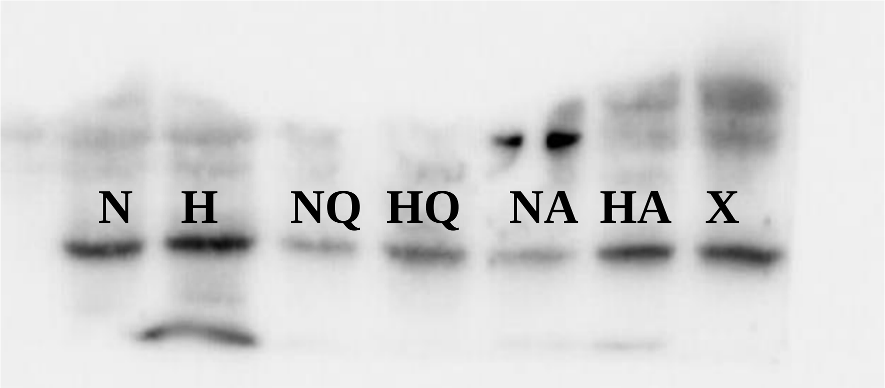

(iv) Time of exposure: 20 s

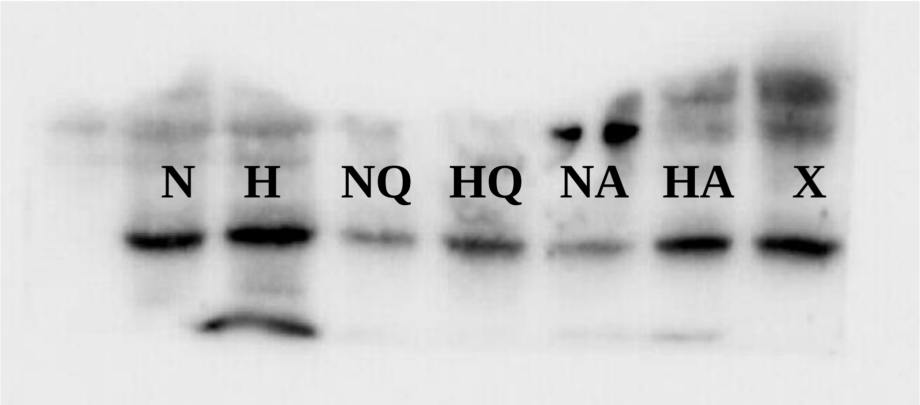

(v) Time of exposure: 30 s

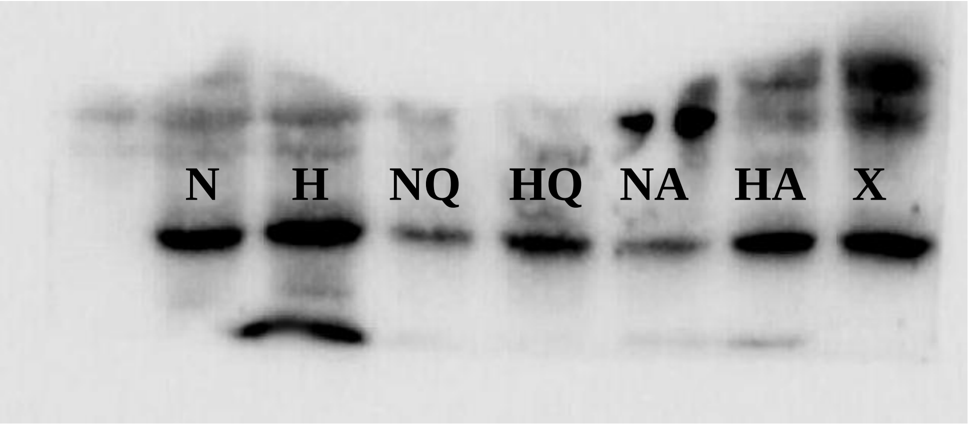

Figure 7

(e) Interleukin-18 (IL-18)

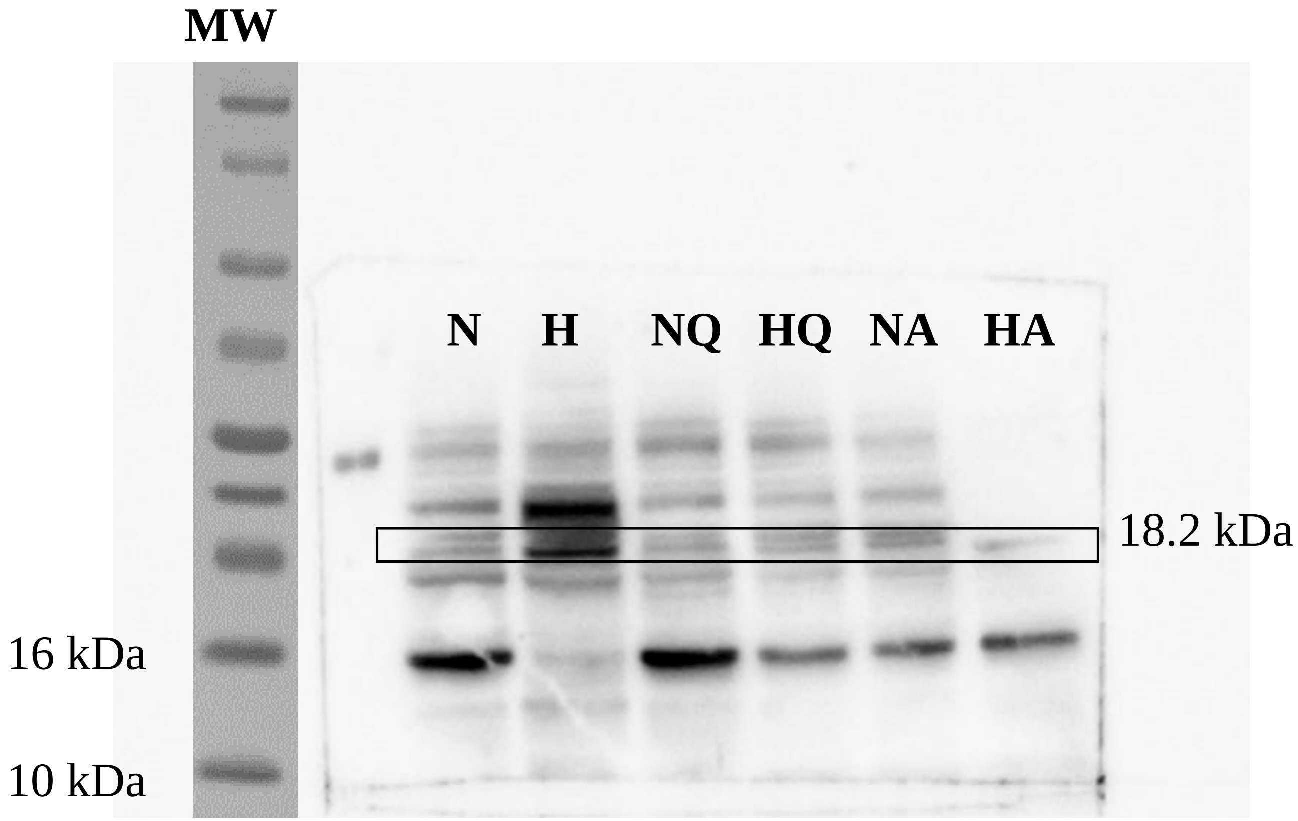

(e) Interleukin-18 (IL-18)

(i) Time of exposure: 5 s

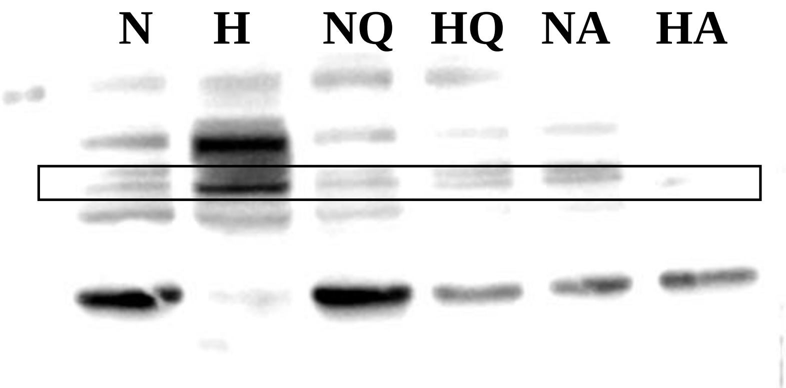

(ii) Time of exposure: 10 s

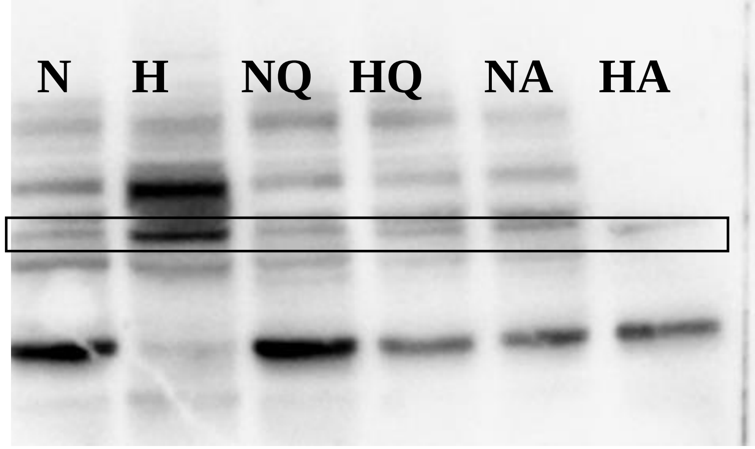

(iii) Time of exposure: 15 s

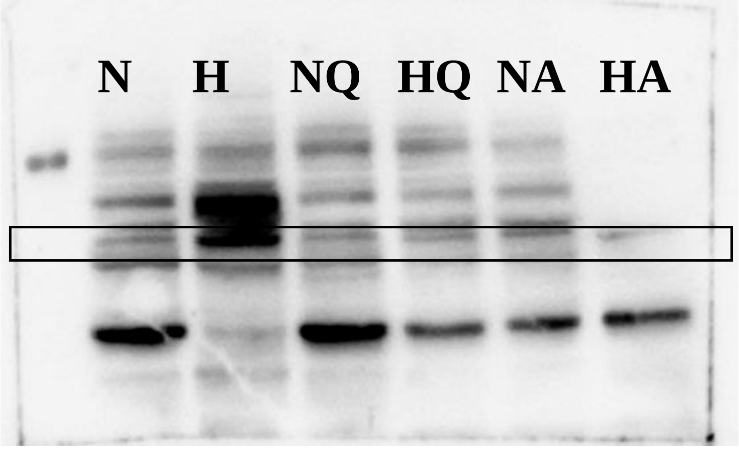

(iv) Time of exposure: 20 s

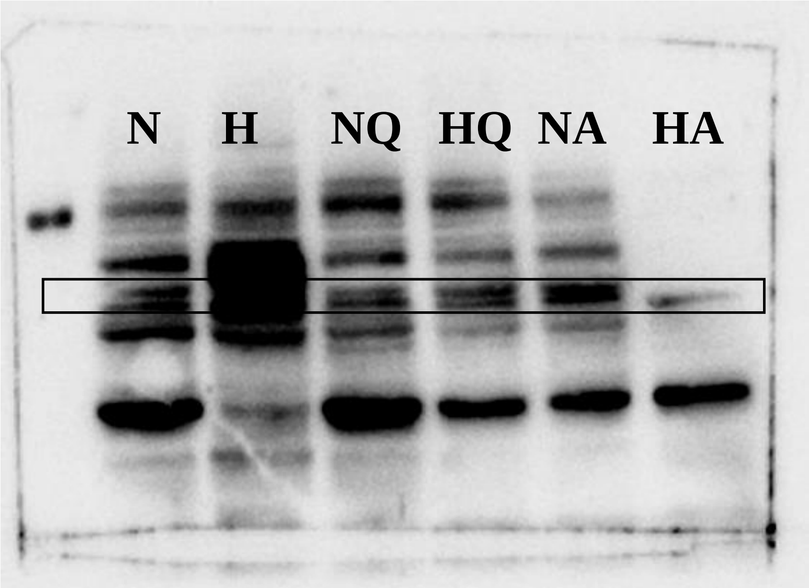

Figure 7

(f) α-Tubulin

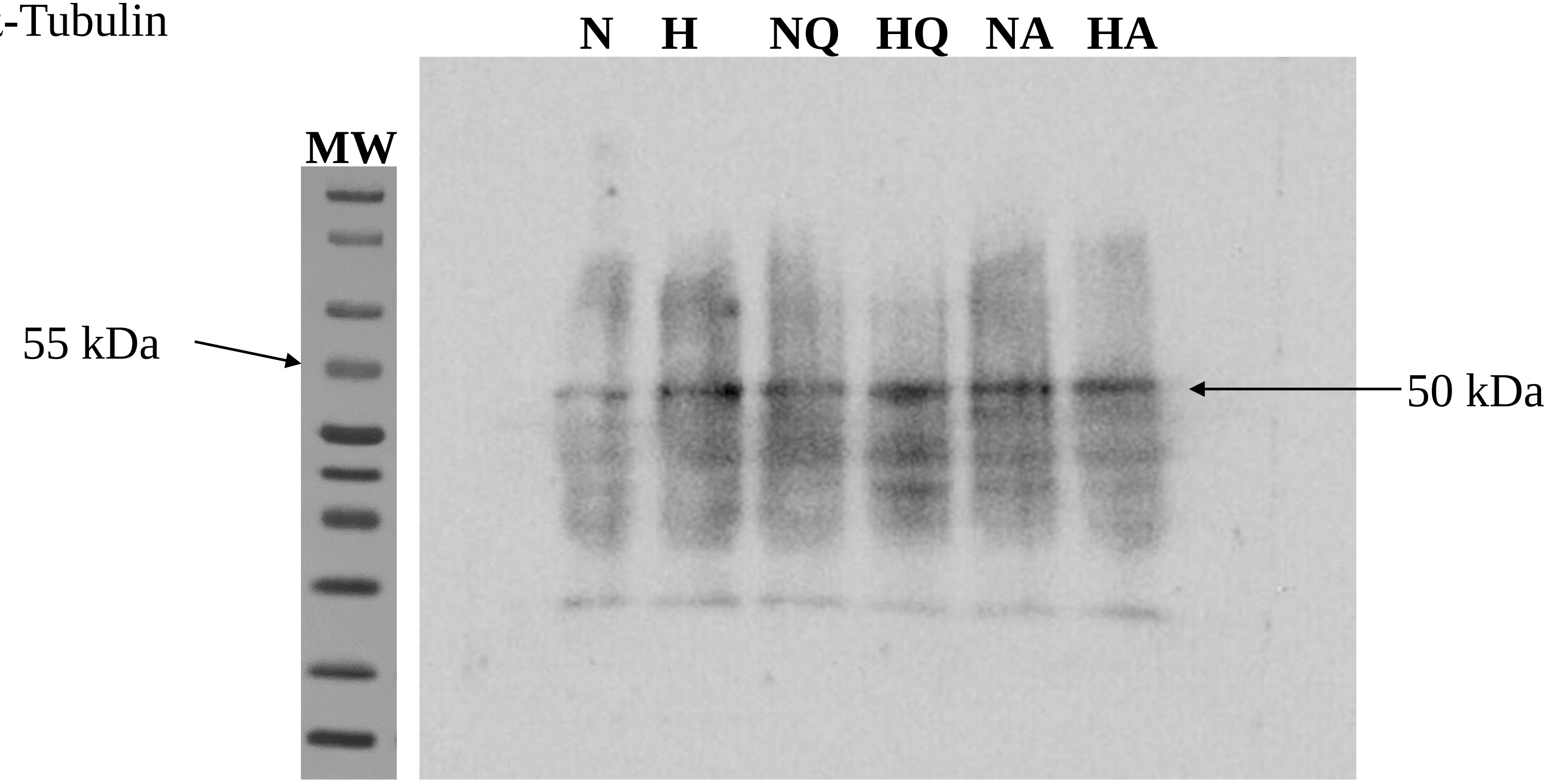

Supplement: Supplementary file 1 — Supplementary Figure S1. [file 41598_2024_58134_MOESM1_ESM.pdf]
